# A Unified Temperature-Dependent Elastoplastic Damage Framework for Concrete from Sub-Zero to Elevated Temperatures

**DOI:** 10.3390/ma19153289

**Published:** 2026-08-03

**Authors:** Ping Gao, Qinglong You, Jinbo Xie, Xi Du, Yungui Pan, Bo Lu, Lixin Chang

**Affiliations:** 1 School of Urban Regeneration, Shanghai Zhongqiao Vocational and Technical University, Shanghai 201514, China; 2 Department of Civil Engineering, School of Environment and Architecture, University of Shanghai for Science and Technology, Shanghai 200093, China; 3 Department of Engineering Geology, Institute of Applied Geosciences, Technical University of Berlin, Ernst-Reuter-Platz 1, 10587 Berlin, Germany; 4 School of Resources and Civil Engineering, Northeastern University, Shenyang 110819, China; 5 College of Intelligent Building Engineering, Shanghai Construction Management Vocational College, Shanghai 201702, China

**Keywords:** concrete constitutive model, thermo-elastoplastic damage model, full-temperature-range constitutive model, reversible low-temperature strengthening, irreversible thermal damage

## Abstract

Concrete exposed to sub-zero and elevated temperatures exhibits strongly non-monotonic mechanical behavior governed by different physical mechanisms. Existing thermo-mechanical constitutive models commonly account for temperature-dependent degradation, but many are formulated for a specific temperature regime, and explicit treatment of reversible freezing-induced strengthening and irreversible high-temperature damage within a single constitutive structure remains limited. This study develops a unified thermo-elastoplastic damage model for concrete over the temperature range from −40 to 800 °C within the framework of irreversible thermodynamics. Plasticity is formulated in the effective-stress space, while compressive damage is driven by the damage energy release rate. Temperature effects are incorporated through evolution laws for compressive strength, elastic modulus, peak strain, and the shape parameters of the ascending and descending branches. Ice-induced strengthening is represented through reversible modifications of stiffness and strength thresholds, whereas high-temperature dehydration and microcracking are represented through irreversible thermal damage. The model was calibrated using published low-temperature compression data for C30–C50 concrete and complete high-temperature stress–strain curves for normal-strength concrete. The normalized curve-shape laws were subsequently assessed using high-strength concrete curves after normalization by their measured peak stress and peak strain, while selected components of the three-dimensional extension were assessed using residual HSC60 true-triaxial data. The calibrated model represented the freezing-point strength valley, sub-zero strengthening and embrittlement, non-monotonic strength evolution at intermediate temperatures, and progressive high-temperature ductilization. Complete high-temperature normal-strength concrete curves were reproduced with R^2^ values of 0.94–0.99, while the normalized multiaxial strength assessment yielded an average relative error of approximately 8%. These results support the internal consistency of the formulation and the limited cross-strength-grade applicability of the normalized curve-shape laws, rather than unrestricted predictive capability. Further independent experiments are required before application beyond the material, moisture, thermal-history, and loading conditions represented by the available datasets.

## 1. Introduction

Concrete is the most widely used structural material in civil and protective engineering, and its service environments are increasingly extending toward both ends of the extreme-temperature spectrum. At the low-temperature end, liquefied natural gas (LNG) storage tanks, cryogenic liquid storage and transportation facilities, cold-region and polar engineering structures, and aerospace structures require concrete to maintain load-bearing and sealing capacities under temperatures far below ambient conditions, even under cryogenic conditions such as LNG temperatures of approximately −165 °C [1,2]. At the high-temperature end, building fires, nuclear containment structures, and metallurgical and energy facilities expose concrete to thermal actions of several hundred degrees Celsius [3,4,5]. In both types of environments, the strength, stiffness, and deformation capacity of concrete vary significantly and non-monotonically with temperature. Therefore, accurate prediction of the mechanical response of concrete over the broad temperature range is a common prerequisite for structural safety design and service-performance assessment.

Research on low-temperature, or cryogenic, concrete has attracted attention since the 1970s and 1980s [6,7] and has recently regained momentum due to the increasing demand from LNG and cold-region engineering [8,9]. Experimental studies generally show that, as temperature decreases, the compressive strength and elastic modulus of concrete increase, whereas the peak strain decreases and brittleness becomes more pronounced [7,10,11,12]. This strengthening is mainly attributed to the freezing of pore water: ice fills and constrains the pore space and participates in load transfer as a load-bearing phase. As a result, the magnitude of strengthening strongly depends on the moisture content and degree of saturation [1,2]. Moreover, this strengthening can largely recover after rewarming, showing a clear reversible character [1]. However, because low-temperature testing devices and loading methods have not yet been fully standardized, the available data remain scattered, and the post-peak descending branch of the stress–strain curve is particularly difficult to capture [9]. This creates substantial difficulty for constitutive model calibration. Moisture content and degree of saturation are therefore essential variables governing the sub-zero mechanical response. However, they are not introduced as independent state variables in the present model because the available dataset does not provide sufficiently detailed moisture histories for their calibration. The resulting temperature-dependent parameter laws should therefore be understood as conditional on the moisture state represented by the source experiments rather than as moisture-independent material laws. It should also be recognized that moisture content and degree of saturation are not secondary variables in the sub-zero response, because they control the amount of pore water available for freezing and load transfer. In the present study, however, moisture content and saturation are not introduced as independent state variables because the available datasets do not provide sufficiently consistent moisture histories for their calibration. The resulting temperature-dependent laws should therefore be understood as conditional on the moisture states represented by the source datasets, rather than as moisture-independent material relations.

In comparison, the behavior of concrete at high temperatures has been more extensively investigated [4,13]. With increasing temperature, evaporation of free water, dehydration and decomposition of hydration products such as C–S–H gel and Ca(OH)_2_, and microcrack propagation induced by thermal incompatibility between aggregates and cement paste generally lead to reductions in strength and stiffness, a pronounced increase in peak strain, and possible explosive spalling [3,5]. In the temperature range of 200–400 °C, however, a non-monotonic recovery of strength is often observed due to moisture migration and re-densification of the gel structure [3,14]. Unlike the reversible strengthening at low temperatures, the degradation caused by dehydration and microcracking at high temperatures is irreversible after cooling and should therefore be regarded as irreversible damage [5,14]. It is thus evident that the temperature domains below and above 0 °C are governed by fundamentally different mechanisms, and the mechanical properties of concrete evolve non-monotonically over the entire temperature range.

At the constitutive level, continuum plasticity–damage theory has become a widely used framework for describing the nonlinear response and post-peak degradation of concrete. Lubliner et al. [15] and Lee and Fenves [16] established the plasticity–damage yield surface that underlies many subsequent concrete models. Ju [17] developed an energy-based elastoplastic damage theory, Faria et al. [18] and Wu et al. [19] formulated damage models driven by energy release rates, and Grassl and Jirásek [20] proposed a coupled damage–plasticity model for concrete failure. For elevated-temperature applications, established approaches have incorporated transient thermal creep, temperature-dependent strength and deformation parameters, thermo-mechanical damage, and design-oriented reduction factors [21,22,23,24,25].

More recent studies have extended these foundations toward moisture-dependent, thermo-plastic, fracture-oriented, and multiscale formulations. Moisture-dependent models have explicitly related thermal strain and load-induced thermal strain to the initial water content [26]. Thermo-mechanically coupled elastoplastic damage formulations have introduced temperature into the free-energy function, yield condition, hardening law, and damage evolution equations [27,28]. Phase-field approaches have been developed to describe fully coupled thermo-mechanical fracture and temperature-dependent material degradation [29]. Multiscale and mesoscopic models have also connected dehydration, aggregate–mortar thermal incompatibility, moisture transport, interfacial damage, and microcracking to the macroscopic response of concrete [30,31].

These studies demonstrate that temperature-dependent degradation, thermo-plasticity, moisture effects, fracture, and multiscale thermal damage have already been addressed through several advanced modeling strategies. However, most of these formulations primarily focus on elevated-temperature deterioration, transient heating, fire-induced fracture, or detailed multiphysics processes. Constitutive treatment of sub-zero strengthening remains comparatively limited, particularly regarding the integration of reversible freezing-induced changes and irreversible high-temperature degradation within the same compact macroscopic framework.

Despite these advances, several issues remain insufficiently resolved when concrete behavior is considered continuously from sub-zero to elevated temperatures. First, many existing thermo-mechanical formulations primarily address elevated-temperature degradation, transient heating, fire-induced fracture, or moisture and pore-pressure evolution [26,27,28,29,30,31]. Sub-zero strengthening is generally treated separately or lies outside their intended temperature range. Second, the dominant mechanisms in the two temperature regimes differ not only in magnitude but also in reversibility. Freezing-induced pore filling and confinement may largely disappear after rewarming, whereas dehydration, thermal incompatibility, and thermally induced microcracking produce persistent deterioration. Representing both effects through an undifferentiated temperature-dependent damage variable may therefore obscure their different physical characteristics. Third, continuous material-level stress–strain data for the same concrete mixture over a broad temperature interval from sub-zero to elevated temperatures remain scarce. The experimental requirements of the two regimes also differ: moisture or saturation is required to activate freezing-induced strengthening, whereas high saturation during heating may intensify pore-pressure buildup and spalling [1,3,26,31].

Accordingly, the research gap addressed in this study is not the absence of temperature-dependent or thermo-mechanical constitutive models. Rather, it is the limited availability of a compact macroscopic framework that retains the same plasticity–damage structure across sub-zero and elevated-temperature regimes, explicitly distinguishes reversible freezing-induced strengthening from irreversible high-temperature degradation, and represents the complete compressive stress–strain response through mechanically interpretable parameter-evolution laws. The proposed model is therefore positioned as a reduced-order constitutive framework rather than a replacement for fully coupled hygro-thermo-mechanical, phase-field, or multiscale models.

To address this specific gap, this study develops a temperature-dependent thermo-elastoplastic damage framework for concrete over the calibrated interval from −40 to 800 °C within the framework of irreversible thermodynamics. Plasticity is formulated in the effective-stress space [17], while compressive damage is driven by the damage energy release rate [18,19]. Temperature enters the formulation through compressive strength, initial elastic modulus, peak strain, the shape parameters of the ascending and descending branches, and an irreversible thermal-degradation description. The same constitutive structure is retained over the considered temperature interval. Freezing-induced strengthening is represented through reversible modifications of the elastic stiffness and strength thresholds, whereas dehydration- and microcracking-induced deterioration at elevated temperatures is represented through irreversible degradation.

Moisture content and degree of saturation are not included as independent state variables. Their effects are represented implicitly through the temperature-dependent parameters calibrated from the source datasets. The resulting parameter laws are therefore conditional on the concrete compositions, moisture states, thermal histories, and testing procedures represented by those datasets.

The framework is calibrated and assessed using several experimental datasets reported in the literature. The low-temperature parameter laws are calibrated using the uniaxial compression data for C30–C50 concrete reported by Li et al. [9], whereas the elevated-temperature laws are calibrated primarily using the complete normal-strength concrete stress–strain curves summarized by Kodur [3]. The normalized curve-shape laws are subsequently assessed using high-strength concrete curves after normalization by their measured peak stress and peak strain. Selected components of the three-dimensional extension are further assessed using the residual HSC60 true-triaxial data reported by He et al. [32].

Because all experimental datasets were obtained from previously published studies and some parameters were determined using the same data employed in the subsequent comparisons, these analyses comprise parameter calibration, internal consistency assessment, and limited cross-dataset assessment rather than fully blind experimental validation. The principal contribution of this study is therefore a thermodynamically consistent and physically interpretable strategy for connecting reversible sub-zero strengthening with irreversible elevated-temperature degradation, rather than a claim of unrestricted predictive capability across arbitrary concrete mixtures, moisture conditions, and loading paths.

## 2. Methodology and Constitutive Formulation

### 2.1. Modeling Strategy and Basic Assumptions

The experimental data available for parameter calibration and model assessment are derived primarily from monotonic uniaxial compression tests. The main constitutive formulation is therefore first presented under uniaxial compression, so that the internal variables and material parameters can be directly related to measurable stress–strain quantities. This uniaxial formulation is a specialization of the energy-based elastoplastic damage framework [18,19], while its extension to three-dimensional tensile and multiaxial stress states is presented in Section 2.7 and assessed in Section 5.

The model is established based on the following assumptions. 

(i) Small-strain kinematics are adopted because the material strains represented by the compression datasets remain sufficiently small for geometric changes to have a secondary influence on the local constitutive response. The additive decomposition of elastic, plastic, and thermal strains can therefore be employed. This assumption is appropriate for material-point calibration and conventional compression analysis, although it would require reconsideration for severe crushing, large deformation, or structural-instability problems.

(ii) Plasticity is formulated in the effective-stress space and updated separately from damage. The effective stress represents the response of the undamaged load-bearing skeleton, whereas damage represents the reduction in effective load-carrying area and elastic stiffness. This separation allows irreversible strain evolution to be evaluated through the plasticity model before the nominal stress is degraded by damage and facilitates an operator-split numerical implementation [16,17,18,19]. Plasticity and damage are nevertheless coupled through the nominal stress relation, the hardening variables, and the damage energy release rate.

(iii) Pre-peak nonlinearity is represented mainly by plastic hardening, whereas compressive damage governs post-peak softening. Under monotonic compression, both plastic deformation and stiffness degradation contribute to the measured nonlinear response. However, the available datasets do not include unloading–reloading paths, which would be required to identify these two contributions independently. A closure assumption is therefore adopted in which the effective stress increases to the compressive strength along the ascending branch, while the descending branch is attributed primarily to compressive damage and stiffness degradation. This treatment enables parameter identification from monotonic stress–strain curves, although it may overestimate the unloading stiffness because pre-peak microcracking is incorporated into the plastic component. This limitation is further discussed in Section 2.5.

(iv) Sub-zero strengthening is treated as reversible under the thermal histories considered in this study. During monotonic cooling, pore-water freezing fills and constrains the pore space and contributes to load transfer. Because the ice phase disappears upon rewarming and the associated strengthening can largely recover, its effect is introduced through reversible modifications of the elastic modulus and strength thresholds rather than through an irreversible damage variable. This assumption applies to single-cooling or isothermal low-temperature loading conditions and does not describe irreversible deterioration accumulated during repeated freeze–thaw cycles.

(v) Elevated-temperature degradation is treated as irreversible thermal damage. Dehydration of cement hydrates, thermal incompatibility between aggregates and cement paste, and thermally induced microcracking produce persistent reductions in stiffness and strength that are not fully recovered after cooling. Their macroscopic effects are therefore represented through irreversible degradation of the temperature-dependent strength and elastic properties. This treatment is phenomenological and does not explicitly resolve moisture transport, chemical decomposition, or individual microcrack evolution.

The proposed formulation follows the general structure of established coupled damage–plasticity models. The Lubliner–Lee–Fenves framework [15,16] provides the multiaxial yield-surface basis and the distinction between tensile and compressive damage. The energy-based formulations of Ju [17], Faria et al. [18], and Wu et al. [19] provide the effective-stress concept and damage evolution driven by energy release rates, while the Grassl–Jirásek model [20] similarly combines permanent plastic deformation with stiffness degradation and strain softening. The present framework does not introduce a fundamentally new generic damage–plasticity coupling architecture. Its specific contribution is the incorporation of temperature-dependent parameter-evolution laws into this established structure and the explicit distinction between reversible freezing-induced strengthening and irreversible elevated-temperature degradation over the considered temperature interval.

### 2.2. One-Dimensional Kinematics

The total strain ϵ is additively decomposed into elastic, plastic, and thermal strain components:

(1) $$\epsilon =\epsilon^{e}+\epsilon^{p}+\epsilon^{\theta }$$

where $\epsilon^{e}$ denotes the recoverable elastic strain, $\epsilon^{p}$ denotes the irreversible plastic strain, and the thermal strain is given by free thermal expansion:

(2) $$\epsilon^{\theta }\left(T\right)=\int_{T_{ref}}^{T}\alpha_{th}\left(T\prime \right)\;dT\prime$$

where $\alpha_{th}$ is the coefficient of thermal expansion and $T_{ref}$ is the reference temperature, usually taken as room temperature. Since all tests used for calibration in this study were conducted under isothermal conditions, with each curve corresponding to a fixed temperature, $\epsilon^{\theta }$ is a constant and does not enter the stress–strain relation at that temperature. Equation (2) is used only to describe thermal deformation in non-isothermal structural analyses. The transient thermal strain induced by heating under sustained load may be additionally incorporated into $\epsilon^{\theta }$ through a coefficient $k_{tr}$ [19,22], but it is not calibrated in this study due to the lack of corresponding experimental data. Therefore, all constitutive relations presented below are formulated in terms of the mechanical strain $\epsilon^{e}+\epsilon^{p}$ at a prescribed temperature T.

### 2.3. Free Energy, Effective Stress, and Constitutive Relations

The Helmholtz free-energy density ψ is introduced. Its elastic part is degraded by compressive damage and supplemented by the plastic hardening energy $\psi^{p}$ [17,18,19]:

(3) $$\psi =\left(1-d^{-}\right)\frac{1}{2}E_{0}\left(T\right){\left(\epsilon -\epsilon^{p}\right)}^{2}+\psi^{p}\left(\kappa_{c}\right)$$

where $E_{0}\left(T\right)$ is the initial elastic modulus at temperature T, and $\kappa_{c}$ is the equivalent plastic strain in compression, serving as a hardening internal variable. Both ψ and $\psi^{p}$ are energy densities per unit volume, with units of $J\;m^{-3}$, equivalently in Pa. When stress and elastic modulus are expressed in MPa, the corresponding energy-density unit is $MJ\;m^{-3}$. The effective compressive stress is defined as the response of the undamaged stiffness to the elastic strain:

(4) $$\bar{\sigma }=E_{0}\left(T\right)\left(\epsilon -\epsilon^{p}\right)$$

Following the Coleman–Noll procedure, the nominal, or Cauchy, stress σ and the damage energy release rate Y are obtained as [18,19]:

(5) $$\sigma =\frac{\partial \psi }{\partial \epsilon }=\left(1-d^{-}\right)\bar{\sigma }$$

(6) $$Y=-\frac{\partial \psi }{\partial d^{-}}=\frac{1}{2}E_{0}\left(T\right){\left(\epsilon -\epsilon^{p}\right)}^{2}=\frac{{\bar{\sigma }}^{2}}{2E_{0}\left(T\right)}\geq 0$$

Equation (5) shows that the nominal stress is obtained by degrading the effective stress through the compressive damage variable, whereas Equation (6) identifies the non-negative damage energy release rate that drives damage evolution. The nominal stress remains the stress quantity used in the equilibrium equations and is directly comparable with the experimentally measured response. The effective stress, by contrast, represents the stress carried by the mechanically undamaged load-bearing skeleton at the prescribed temperature.

The effective-stress formulation is particularly suitable for the present application because it separates three effects that would otherwise be mixed in the nominal-stress space: plastic deformation of the load-bearing skeleton, reversible temperature-induced changes in stiffness and strength thresholds, and irreversible stiffness degradation caused by damage. If the plastic yield condition were written directly in terms of nominal stress, the stress entering the yield function would decrease as damage develops. Consequently, the apparent plastic strength and flow response would depend implicitly on the current damage level, making it difficult to distinguish plastic yielding from damage-induced stress reduction. This difficulty is especially relevant here because sub-zero strengthening is represented through reversible changes in stiffness and strength, whereas elevated-temperature deterioration and post-peak softening are represented through irreversible degradation.

By defining plasticity in the effective-stress space, the yield condition and plastic flow describe the response of the current undamaged skeleton, while the nominal stress is obtained afterward through damage degradation. The plastic return-mapping step can therefore be performed using the effective stress and the temperature-dependent strength threshold, followed by an independent update of the damage variable from the damage energy release rate. This operator-split structure avoids repeated modification of the plastic yield surface solely because of stiffness degradation and provides a clearer numerical distinction between irreversible plastic strain and loss of elastic load-carrying capacity.

The use of effective stress does not imply that plasticity and damage are physically independent. They remain coupled through the strain decomposition, the nominal stress relation, the hardening variables, and the damage energy release rate. Rather, the effective-stress formulation provides a convenient thermodynamic and computational framework for separating their respective contributions during constitutive integration. Because this separation is calibrated primarily from monotonic compression data, its accuracy under cyclic unloading–reloading paths still requires additional experimental assessment.

### 2.4. Dissipation, Plasticity, and Damage Evolution

Following the energy-based coupled elastoplastic damage theory [17], plasticity is defined in the effective stress space, and the mechanical dissipation (D) can be reduced to the sum of plastic dissipation and damage dissipation:

(7) $$D=\left(\bar{\sigma }{\dot{\epsilon }}^{p}-q{\dot{\kappa }}_{c}\right)+\left(Y{\dot{d}}^{-}\right)\geq 0$$

where the hardening force is defined as

(8) $$q=\frac{\partial \psi^{p}}{\partial \kappa_{c}}$$

The two terms are individually non-negative, thereby ensuring the thermodynamic admissibility of the entire process:

(9) $$\bar{\sigma }{\dot{\epsilon }}^{p}-q{\dot{\kappa }}_{c}\geq 0,\;\;Y{\dot{d}}^{-}\geq 0$$

It should be noted that the plastic work obtained strictly from the Coleman–Noll procedure is the nominal plastic work,

(10) $$\sigma {\dot{\epsilon }}^{p}=\left(1-d^{-}\right)\bar{\sigma }{\dot{\epsilon }}^{p}$$

which differs from the effective-stress plastic work adopted in Equation (7) only by the non-negative factor $1-d^{-}$. This difference does not affect the non-negativity of dissipation. The purpose of adopting the effective-stress formulation [17] is to allow the plastic flow and damage update to be solved independently through operator splitting, thereby improving numerical robustness.

The yield condition is defined in the effective stress space. For uniaxial compression, the yield function is written as

(11) $$F=\left|\bar{\sigma }\right|-{\bar{c}}_{c}\left(\kappa_{c}\right)\leq 0$$

where ${\bar{c}}_{c}\left(\kappa_{c}\right)$ is the effective compressive cohesion or the current compressive strength. It hardens with $\kappa_{c}$, increases to the peak effective strength $f_{c}$, and then remains saturated. The plastic flow rule and the Kuhn–Tucker loading-unloading conditions are given by

(12) $${\dot{\epsilon }}^{p}=\dot{\lambda }\frac{\partial F}{\partial \bar{\sigma }}=\dot{\lambda }\;sign\left(\bar{\sigma }\right),\;\;\dot{\lambda }\geq 0,\;F\leq 0,\;\dot{\lambda }F=0$$

where $\dot{\lambda }$ is the plastic multiplier. The compressive damage variable $d^{-}$ is driven by the energy release rate Y. Its threshold is irreversible and shifts with temperature; sub-zero temperature strengthening increases the threshold, with $r_{0}^{-}\left(T\right)\propto k_{f}\left(T\right)$, thereby ensuring ${\dot{d}}^{-}\geq 0$ [18,19]. The specific one-dimensional form of this evolution law is determined by the skeleton curve in the next section.

### 2.5. Uniaxial Compressive Skeleton Curve and Damage Calibration

The two-parameter curve proposed by Guo [33] and adopted in GB 50010-2010 [34] is used as the skeleton curve. Let the dimensionless strain be $x=\epsilon /\epsilon_{p}\left(T\right)$ and the dimensionless stress be $y=\sigma /f_{c}\left(T\right)$, where the peak stress $f_{c}\left(T\right)$ already includes the contributions of thermal damage $d_{\theta }$ and sub-zero temperature strengthening:

(13) $$y=\left\{\begin{array}{ll} \alpha_{a}\left(T\right)x+\left(3-2\alpha_{a}\left(T\right)\right)x^{2}+\left(\alpha_{a}\left(T\right)-2\right)x^{3}, & 0\leq x\leq 1,\\ \frac{x}{\alpha_{d}\left(T\right){\left(x-1\right)}^{2}+x}, & x>1. \end{array}\right.$$

The ascending-branch parameter $\alpha_{a}=E_{0}\epsilon_{p}/f_{c}\geq 1$ is the ratio between the initial tangent modulus and the peak secant modulus, and it controls the pre-peak curvature. The descending-branch parameter $\alpha_{d}$ controls the steepness of softening. Owing to the limitation of monotonic tests without unloading, a closure assumption is introduced to separate plasticity from damage: the effective stress is assumed to increase monotonically to $f_{c}$ along the ascending branch and then remain saturated. Thus, all pre-peak nonlinearity is attributed to plastic hardening, with $d^{-}=0$, whereas all post-peak softening is attributed to compressive damage. Accordingly, the post-peak damage can be directly obtained from the nominal curve:

(14) $$d^{-}\left(x\right)=1-y\left(x\right)=\frac{\alpha_{d}{\left(x-1\right)}^{2}}{\alpha_{d}{\left(x-1\right)}^{2}+x},\;\;x>1$$

A larger $\alpha_{d}$ indicates faster growth of $d^{-}$, steeper softening, and greater brittleness. This assumption gives $\alpha_{d}$ a clear damage-related meaning and directly converts the calibration of the descending branch into damage calibration. Its cost is that the stiffness degradation induced by pre-peak microcracking is incorporated into plasticity; therefore, the residual stiffness would be overestimated under unloading–reloading paths. A stricter separation requires cyclic unloading data, as discussed in Section 6.3. It is worth noting that the ascending branch in Equation (13) is a special case of the more general polynomial $y=\alpha_{a}x+Bx^{2}+Cx^{n}$ with the exponent n=3 [33]. On this basis, Section 5.2 extends the same skeleton-curve family to multiaxial equivalent uniaxial curves. Therefore, the present model is mainly intended for monotonic compressive loading paths, and its use for cyclic or unloading–reloading simulations requires additional stiffness-degradation calibration.

### 2.6. Temperature-Dependent Parameters

The temperature effect is carried by four material parameters: the compressive strength $f_{c}\left(T\right)=k_{f}\left(T\right)f_{c0}$, the peak strain $\epsilon_{p}\left(T\right)=k_{\epsilon }\left(T\right)\epsilon_{p0}$, the ascending-branch parameter $\alpha_{a}\left(T\right)$, and the descending-branch parameter $\alpha_{d}\left(T\right)$. The initial modulus is accordingly given by $E_{0}\left(T\right)=k_{E}\left(T\right)E_{0}$, and it remains consistent with $\alpha_{a}=E_{0}\epsilon_{p}/f_{c}$. Here, $k_{f}$, $k_{\epsilon }$, and $k_{E}$ are normalized factors with respect to their room-temperature values. Since the mechanisms above and below 0 °C are different, the relevant factors are defined piecewise around the freezing point. The strength factor in the sub-zero regime is expressed with a freezing-point transition as follows:

(15) $$k_{f}\left(T\right)=\left\{\begin{array}{ll} 1+\left(k_{f0}-1\right)\frac{T_{ref}-T}{T_{ref}}, & 0\leq T\leq T_{ref},\\ k_{f0}+B\sqrt{-T}, & T<0. \end{array}\right.$$

where $k_{f0}$ is the valley value at 0 °C and B is the sub-zero recovery coefficient. In the sub-zero regime, the peak-strain factor is taken as linear, whereas in the high-temperature regime it is taken as quadratic. The parameters $\alpha_{a}\left(T\right)$ and $\alpha_{d}\left(T\right)$ are determined from the curve shapes at each temperature. The parameter values are calibrated separately from the low-temperature and high-temperature tests in Section 3 and Section 4, respectively.

The temperature functions adopted here are mechanism-informed empirical representations rather than unique relations derived directly from pore-scale thermodynamics. Their functional forms were selected according to four criteria: consistency with the observed trend, satisfaction of the room-temperature normalization condition, preservation of physically admissible parameter values, and use of the minimum number of fitting coefficients supported by the available data.

The square-root form used for the sub-zero strength factor represents a relatively rapid recovery of strength immediately below the freezing point, followed by progressively smaller increments with further cooling. This trend is qualitatively consistent with the progressive freezing of pore water from larger pores to smaller pores and the depression of the freezing temperature in confined pores. However, the square-root dependence should not be interpreted as a direct derivation from the pore-freezing mechanism; it is a parsimonious empirical approximation to the measured freezing-point transition.

Linear functions are adopted for the sub-zero elastic-modulus and peak-strain factors because the available data cover a relatively narrow temperature interval and display approximately linear trends. Only five temperature levels are available for each concrete grade, and higher-order expressions would introduce additional parameters without sufficient experimental support. The linear forms therefore describe the first-order temperature dependence while reducing the risk of overfitting.

For the elevated-temperature peak-strain factor, a quadratic function of the normalized temperature is used because the measured peak strain increases slowly at lower temperatures and more rapidly at higher temperatures. A linear function cannot represent this accelerating trend, whereas the quadratic form is the lowest-order polynomial that captures the observed curvature while automatically satisfying the room-temperature normalization condition. The descending-branch parameter is represented by an exponential decay because it remains positive and decreases approximately monotonically with temperature. The exponential form describes a nearly constant fractional rate of reduction and avoids the negative values that may result from linear extrapolation. These functional forms are therefore selected for mathematical parsimony and consistency with the available observations, rather than as universal.

### 2.7. Relation to the Three-Dimensional Plastic-Damage Framework

Equations (1)–(11) constitute a specialization of the energy-based elastoplastic damage constitutive framework [18,19] under uniaxial compression. Its extension to three dimensions is direct and natural. The effective stress tensor is spectrally decomposed into tensile and compressive parts:

(16) $$\bar{\sigma }={\bar{\sigma }}_{t}+{\bar{\sigma }}_{c}$$

Tensile and compressive damage variables, $d_{t}$ and $d_{c}$, are then introduced separately, yielding the nominal stress tensor:

(17) $$\sigma =\left(1-d_{t}\right){\bar{\sigma }}_{t}+\left(1-d_{c}\right){\bar{\sigma }}_{c}$$

The yield surface adopts the Lubliner–Lee–Fenves form, including the parameter α determined by the biaxial-to-uniaxial compressive strength ratio, the parameter γ determined by the tensile-to-compressive meridian ratio, and the coupling term β between tensile and compressive cohesion; its explicit form is given in Equation (29). A non-associated flow rule is used [15,16,17,20]. In an implicit finite-element implementation, the three-dimensional constitutive update can follow a conventional elastic predictor–plastic corrector procedure. The trial effective stress is first evaluated using the temperature-dependent elastic parameters. If the trial state violates the yield condition, the plastic multiplier and hardening variables can be updated through a backward-Euler return-mapping algorithm, after which the tensile and compressive damage variables are updated and the nominal stress is reconstructed. A consistent algorithmic tangent is preferable for global Newton iterations, although it is generally nonsymmetric because of the non-associated flow rule. The principal additional computational operations are the spectral decomposition of the effective stress tensor and the solution of the local return-mapping equations, while the proposed temperature functions require only scalar evaluations. A complete finite-element implementation and systematic convergence assessment are beyond the scope of the present study. It should be emphasized that the temperature functions calibrated in this study, namely $k_{f}$, $k_{\epsilon }$, $\alpha_{a}$, $\alpha_{d}$, and $k_{E}$, scale the basic quantities $f_{c}$, $\epsilon_{p}$, and $E_{0}$, which are required by any of the above three-dimensional models. Therefore, the full-temperature-range parameter-evolution laws proposed in this study are independent of the specific constitutive form and can be directly transferred into a three-dimensional framework.

The scope of the present study is therefore limited to the complete uniaxial formulation, temperature-dependent parameter calibration, and assessment of selected components of the three-dimensional extension. Section 5 examines the multiaxial compressive strength surface and equivalent uniaxial stress–strain response using published triaxial data. These comparisons assess the compatibility of the proposed temperature-dependent laws with the three-dimensional framework but do not constitute verification of a complete finite-element implementation.

## 3. Low-Temperature Calibration and Consistency Assessment

### 3.1. Experimental Dataset and Scope of Applicability

The low-temperature regime was calibrated using the uniaxial compression tests of Li et al. [9] on ordinary concrete of four strength grades, namely C30, C35, C40, and C50. The specimens were prisms of 100 mm × 100 mm × 300 mm. Each grade was tested at five temperatures, 20, 0, −20, −30, and −40 °C, with three specimens per grade per temperature, giving 60 specimens in total. P.O 42.5 cement was used, and the moisture content measured after oven drying decreased slightly with strength grade. For each condition, Li et al. reported the mean compressive strength *f*_c_, the mean elastic modulus *E* (secant modulus over 0.2–0.6 *f*_c_), and the mean peak strain *ε*_p_. These reported mean values are adopted here as the calibration targets. As noted in previous low-temperature studies [8,11], both strength and stiffness increase under sub-zero conditions, although the testing methods are not yet standardized and the reported data remain comparatively scattered.

Two characteristics of the raw stress–strain curves must be recognized before they are used. First, the recorded strain is an apparent strain. Deformation was measured from the displacement of the testing machine [9], so it includes the compliance of the loading frame and the seating deformation at the platens. Taken literally, the apparent peak strain reaches about 20 × 10^−3^, which is incompatible with the reported modulus of about 22 GPa: the corresponding ratio

(18) $$\alpha_{a}=\frac{E_{0}\varepsilon_{p}}{f_{c}}$$

would be of order ten, whereas physically reasonable values are about 1.5–2.5 [33,34], where $\alpha_{a}$ is dimensionless, because $E_{0}$ and $f_{c}$ have the same stress unit, while $\varepsilon_{p}$ is dimensionless. The normalized curves also display a nonphysical upward concavity, with a soft toe produced by platen seating. Second, the descending branch is unavailable: owing to the limited stiffness of the testing machine, only the stable ascending branch could be recorded [9], because the elastic strain energy stored in the soft frame was released abruptly after the peak load.

Accordingly, the data are used at two levels. The compressive strength is obtained from the measured load and is independent of the strain measurement; it is therefore adopted directly. The material modulus and peak strain are taken from the reported mean values, which lie in the physically reasonable range of about 1.9–4.1 × 10^−3^, increase with strength grade, and are mutually consistent with the reported strength and ascending parameter through

(19) $$\varepsilon_{p}=\frac{\alpha_{a}f_{c}}{E_{0}}$$

**Figure 1:**
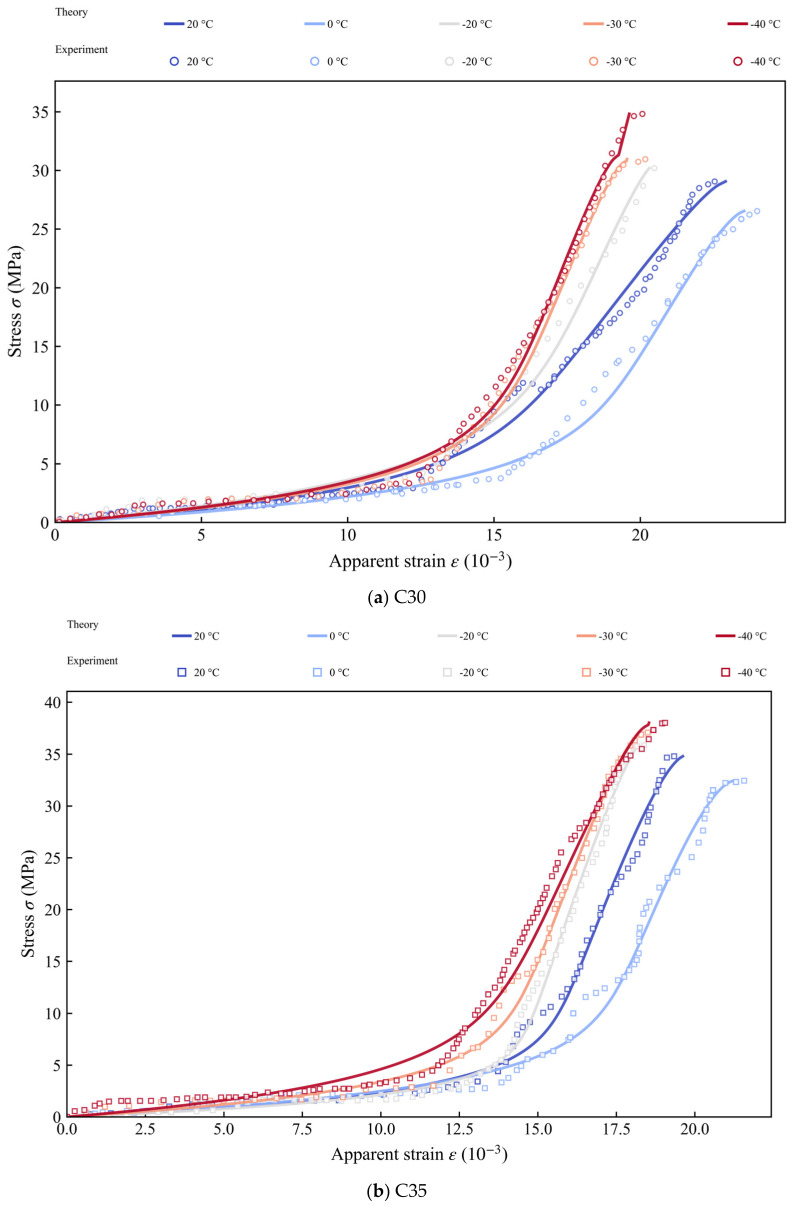

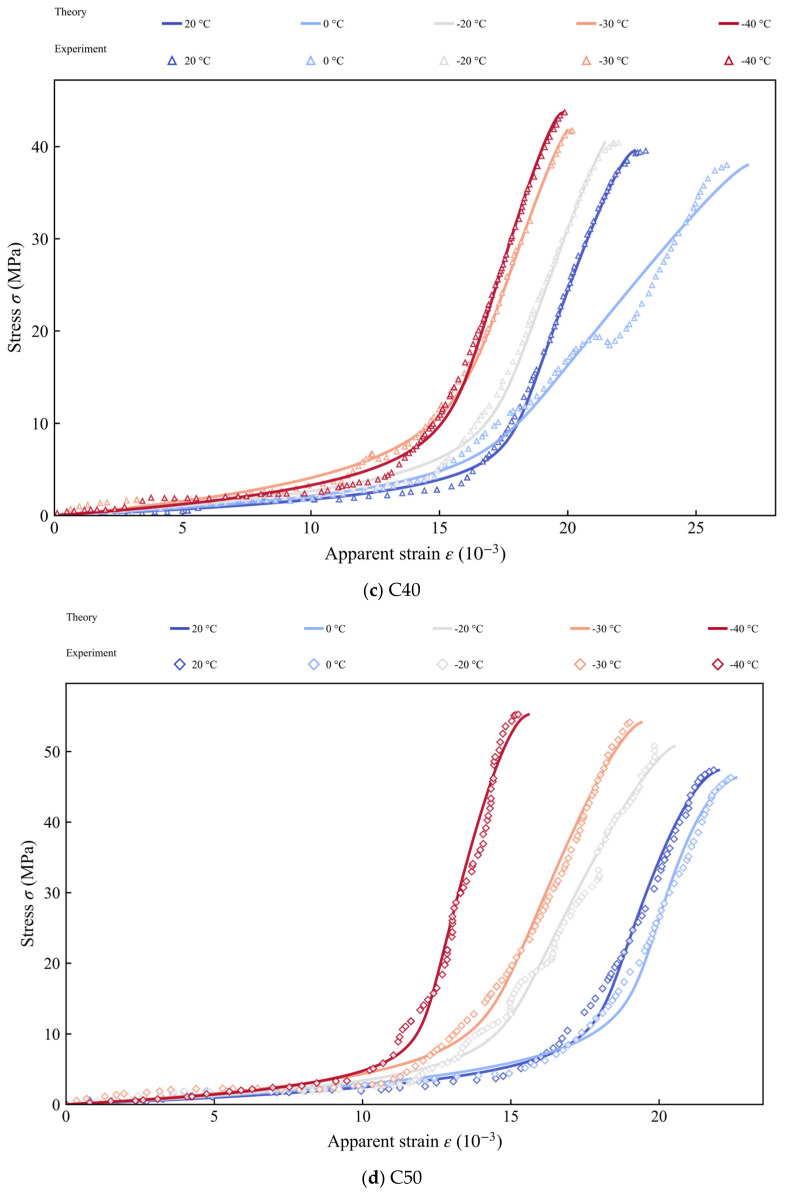

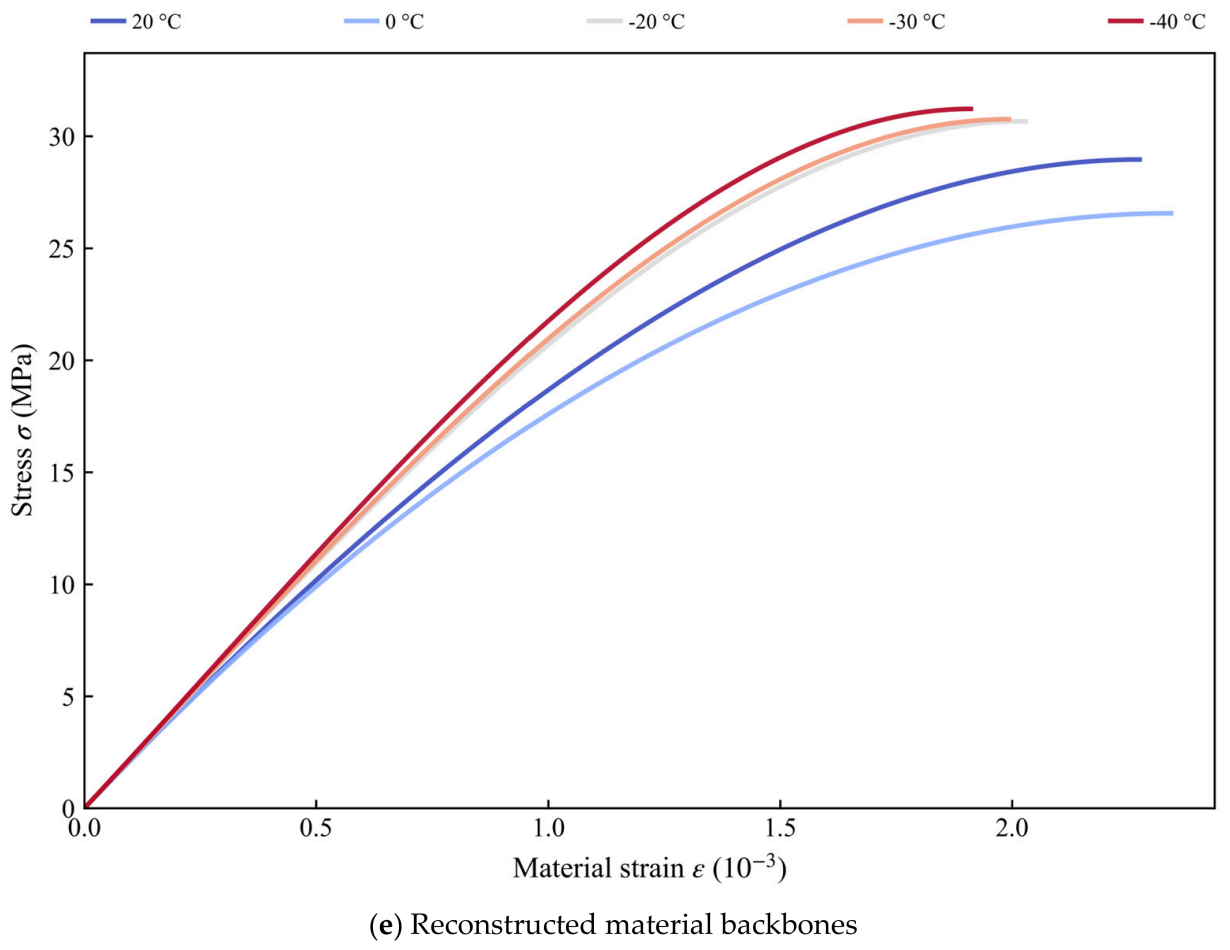
(**a**–**d**) Experimental (symbols) versus theoretical (lines) stress–strain curves in the as-measured (apparent) space for the four design strengths C30, C35, C40, and C50 at 20 to −40 °C; the theory is the material model carried through the fitted machine compliance and platen seating. (**e**) Reconstructed material backbones for C30, with material peak strains of about 2 × 10^−3^—roughly eight times smaller than the apparent strain—showing the freezing-point softening at 0 °C and the sub-zero strengthening.

To confirm that these material-level quantities are consistent with the recorded curves, the apparent response is reproduced by a forward measurement model in which the machine artifacts are superposed on the material backbone (*ε*_app_(*σ*) = *ε*_mat_(*σ*) + *C*_m_ *σ* + *s* [1 − exp(−*σ*/*σ*_s_)], where *ε*_mat_(*σ*) is the skeleton of Equation (13) evaluated with the reported (*f*_c_, *ε*_p_, *α*_a_), *C*_m_ is the frame compliance, and the last term represents seating closure). Fitting only the two machine parameters per curve, with the material backbone held fixed, reproduces the apparent curves of all four grades to within about 5% (Figure 1a–d). For C30, the apparent peak strain of about 20 × 10^−3^ decomposes into roughly 2 × 10^−3^ of material strain, 7 × 10^−3^ of frame compliance, and 14 × 10^−3^ of seating; the recorded strain is therefore dominated by the testing setup rather than by the specimen. This confirms the approximately eightfold difference between apparent and material strain and justifies using the reconstructed material backbones (Figure 1e) for calibration. Because the descending branch is absent, the compressive softening/damage parameter *α*_d_ cannot be calibrated directly and is assigned a prior form (Equation (13)). The validation targets in this section are therefore the temperature evolution of strength, modulus, and peak strain, together with their consistency across grades, rather than material-level curve reproduction.

### 3.2. Calibration of Low-Temperature Parameters

**Figure 2:**
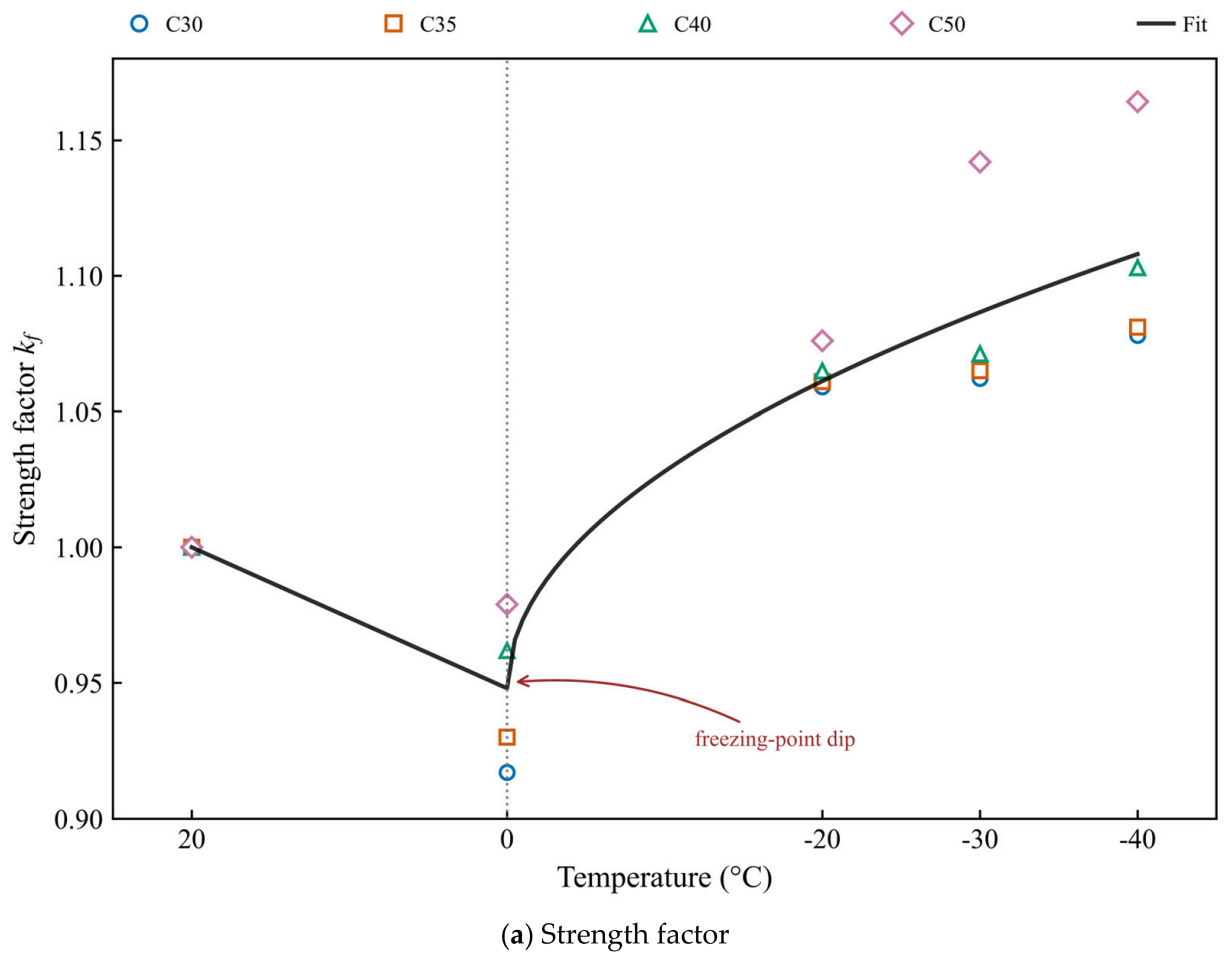

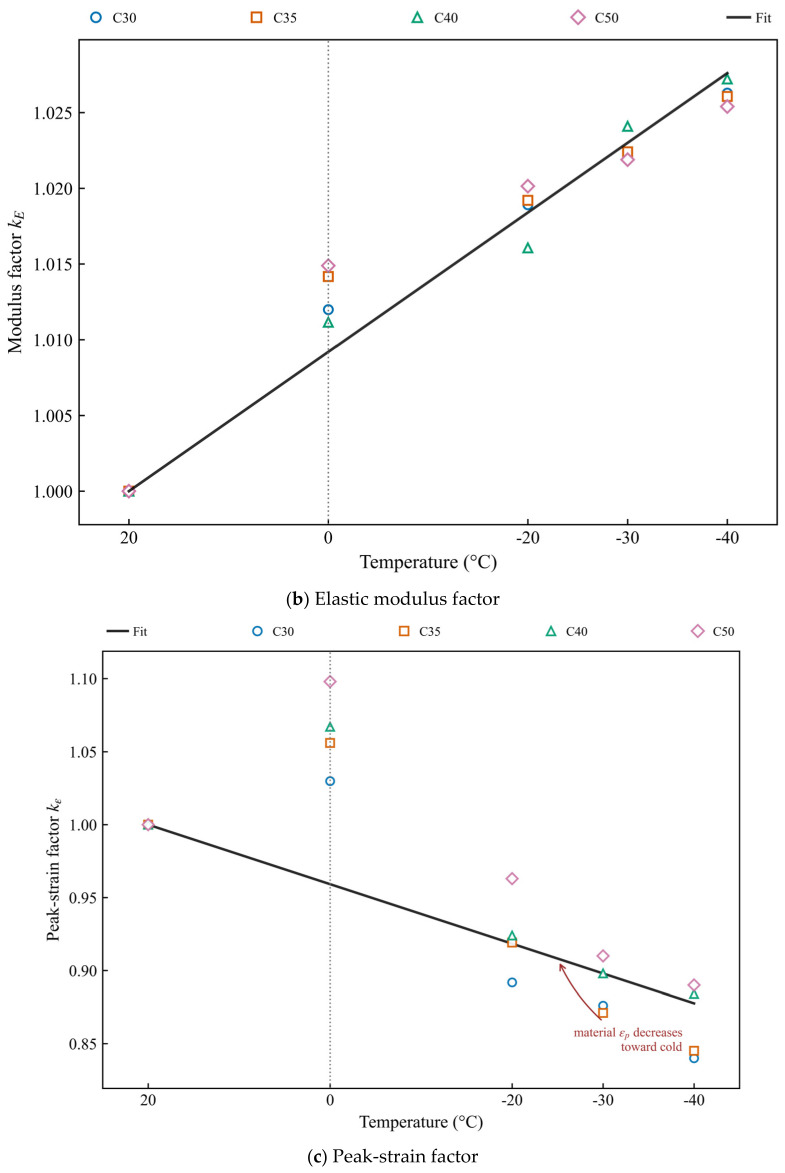
Parameter-evolution laws in the low-temperature regime based on the pooled C30–C50 data.

The reported values of the four grades were normalized by their 20 °C counterparts and pooled for least-squares fitting according to the proposed parameter-evolution laws (Figure 2). The strength factor *k*_f_(*T*) was fitted with the freezing-point form of Equation (15), yielding *k*_f0_ = 0.948 and *B* = 0.0253 °C^−1/2^, with a pooled coefficient of determination *R*^2^ = 0.85. At −40 °C,

(20) $$k_{f}=\;0.948\;+\;0.0253\;\times \;\sqrt{40\;}=\;1.11$$

which agrees with the measured grade means, rising from about 1.08 for C30 to about 1.16 for C50; the strengthening increases progressively with strength grade, confirming the observation of Li et al. [9]. The elastic-modulus factor *k*_E_ was fitted linearly as

(21) $$k_{E}=1+\frac{0.046\;\left(T_{ref}-T\right)}{100}$$

with *R*^2^ = 0.95 (Figure 2b); at −40 °C the modulus increases by only about 2.7%, much less than the strength, consistent with the conclusion of Lee et al. [10] that the modulus rises with cooling but at a lower rate than strength. The material peak-strain factor, obtained from the reconstructed values, was fitted linearly as

(22) $$k_{\varepsilon }\approx 1+0.00204\;\left(T-20\right)$$

giving about 0.88 at −40 °C, so that the material becomes less deformable and more brittle as temperature decreases (Figure 2c). The linear forms in Equations (21) and (22) were selected because the measured modulus and reconstructed material peak strain exhibit approximately first-order trends over the limited interval from −40 to 20 °C. Given the five available temperature levels, higher-order functions were not considered sufficiently constrained by the data. The reported data show a mild local maximum at 0 °C (*k*_ε_ ≈ 1.03–1.10 across grades) before decreasing below 0 °C; this minor ductility maximum coincides with the freezing-point strength valley and reflects the softened, not-yet-frozen state near 0 °C. For consistency with the unified full-range formulation, the sub-zero *k*_ε_ is retained in the linear form of Equation (22), which smooths over this minor feature. The ascending-branch parameter *α*_a_ = *E*_0_*ε*_p_/*f*_c_ decreases from about 1.7–2.1 near 0 °C to about 1.3–1.4 at −40 °C, indicating a more linear ascending branch and reduced pre-peak plastic deformation. Because no low-temperature descending-branch data are available, $\alpha_{d}(T)$ cannot be calibrated directly. The following physically motivated prior is therefore introduced to predict the cold-regime post-peak response:

(23) $$\alpha_{d}\left(T\right)=\alpha_{d0}{\left(\frac{\alpha_{a}\left(20\right)}{\alpha_{a}\left(T\right)}\right)}^{1.5},\;\;\;\;\;\;\;\;\;T<20\;{}^{\circ}C$$

so that *α*_d_ increases by about 1.4 times its room-temperature value at −40 °C. This trend is supported by the original observation that the failure mode changes from local cracking at 20 °C to global splitting and cone-shaped fragmentation at −30 and −40 °C [9], indicating a marked increase in brittleness. Equation (23) is a predictive extrapolation rather than a calibrated relationship. Its functional form reflects the assumption that the increasingly linear ascending branch and the observed change toward splitting and fragmentation at lower temperatures correspond to faster post-peak damage evolution. Because complete low-temperature descending branches are unavailable, both the power-law form and the exponent 1.5 remain unvalidated and should be recalibrated when suitable post-peak experimental data become available.

The power-law form and its exponent of 1.5 are not identified statistically from descending-branch data. They constitute a physically motivated prior that links the increasing linearity of the ascending branch to the observed increase in low-temperature brittleness. Equation (23) should therefore be interpreted as a provisional extrapolation to be recalibrated when complete low-temperature post-peak curves become available. Future validation of Equation (23) requires complete post-peak compression tests at sub-zero temperatures under closed-loop displacement or strain control. Direct specimen deformation should be measured using extensometers, local LVDTs, or digital image correlation to exclude machine compliance and platen-seating effects. Tests should be conducted on the same concrete mixture and under controlled moisture conditions at several temperatures between 20 and −40 °C. The measured descending-branch slope, residual stress, damage evolution, and fracture energy could then be compared with the values predicted by Equation (23), and the exponent and temperature dependence of $\alpha_{d}$ could be recalibrated if necessary. Because only condition-level mean values were available from the source study, specimen-level standard deviations and confidence intervals could not be reconstructed, and the residual analysis is limited to deviations between the fitted functions and the reported mean values. Accordingly, the reported $R^{2}$ values quantify only the agreement between the fitted functions and the available mean values and should not be interpreted as a complete uncertainty assessment. The pooled normalized relationships should be interpreted as common trends for the C30–C50 concretes investigated in the source study, rather than as mixture-independent material laws. Aggregate type, water-to-cement ratio, cement composition, air content, and initial moisture condition may alter the pore-size distribution, interfacial transition zones, amount of freezable water, and internal restraint provided by ice, thereby affecting both the magnitude and temperature dependence of sub-zero strengthening. Because these variables were not varied independently in the available dataset, their individual effects cannot be separated from the present calibration. Consequently, the normalized relationships require mixture-specific assessment or recalibration before being applied to concretes with substantially different constituent materials, pore structures, or moisture conditions.

### 3.3. Validation Results and Discussion

The low-temperature strength law reproduces the normalized data of all four grades, and this consistency across grades constitutes an internal validation of the cold regime. Li et al. [9] reported that, under identical conditions, the strength variation depends only on temperature and is independent of strength grade; this behavior is represented by the unified *k*_f_(*T*). The model captures the freezing-point valley near 0 °C: from 20 to 0 °C the strength decreases by about 5% as the not-yet-frozen pore water provides no load-bearing capacity, whereas below 0 °C the strength recovers, the stiffness increases, the peak strain decreases, and the response becomes more brittle. The underlying micromechanism is discussed in Section 6.1, where this sub-zero strengthening is treated as a reversible effect and is incorporated into *E*_0_ and the strength threshold rather than into damage, consistent with its recovery upon rewarming.

The forward measurement model further validates the calibration at the curve level: for all four design strengths and every temperature, the modeled curves reproduce the measured (apparent) stress–strain responses (Figure 1a–d), confirming that the reconstructed material backbones, illustrated for C30 in Figure 1e, are consistent with the data across the full range of strength grades. Nevertheless, because the machine parameters are fitted nuisance quantities and the post-peak branch is missing, the cold-regime validation remains weaker than the high-temperature validation of Section 4, which reproduces complete material-level stress–strain curves including post-peak softening. In particular, the cold-end *α*_d_ is a physically based prior (Equation (23)) rather than a directly calibrated parameter.

## 4. High-Temperature Calibration and Cross-Grade Assessment

### 4.1. Experimental Dataset

**Figure 3:**
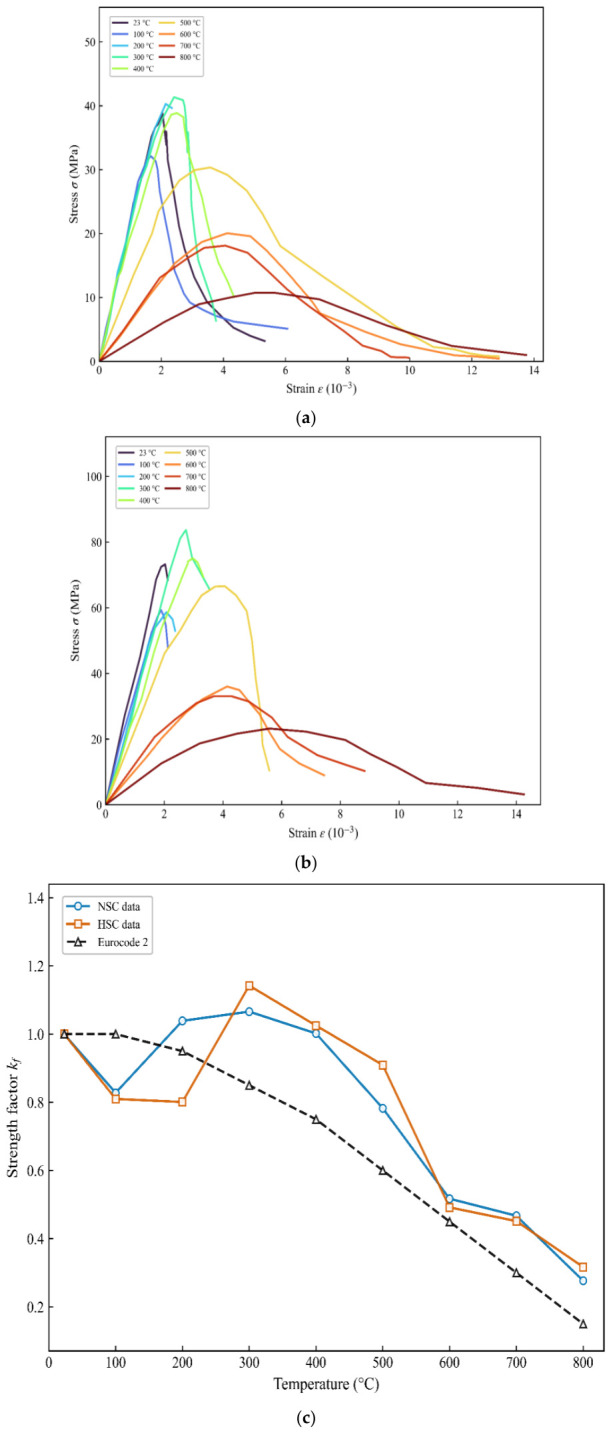

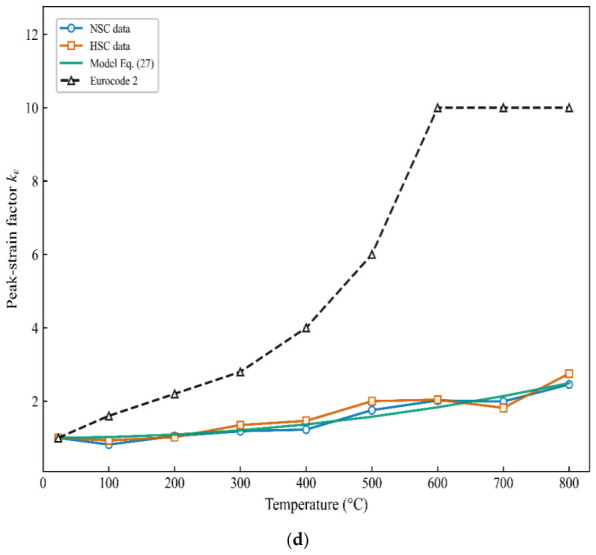
Complete high-temperature stress–strain curves of Kodur’s (**a**,**b**) HSC from 23 to 800 °C, including post-peak softening, and comparison of the (**c**) strength factor and (**d**) peak-strain factor of NSC and HSC with Eurocode 2 (siliceous aggregate). The model peak-strain law (Equation (27)) is shown in (**d**).

For the high-temperature regime, this study uses the uniaxial compressive stress–strain curves of normal-strength concrete (NSC) and high-strength concrete (HSC) reported in the review by Kodur [3]. The temperatures considered are 23, 100, 200, 300, 400, 500, 600, 700, and 800 °C, and the room-temperature compressive strengths are approximately 39 MPa and 73 MPa for NSC and HSC, respectively. Unlike the low-temperature dataset, these curves are based on material strain, with peak strains ranging from about 2 × 10^−3^ to 5.6 × 10^−3^, which is physically reasonable. Moreover, most temperatures include a complete descending branch. These data can therefore be used directly for the calibration and validation of full material-level stress–strain curves, including post-peak softening. The complete curves of the two concretes, together with the temperature dependence of *k*_f_ and *k*_ε_, are compared with Eurocode 2 [25] in Figure 3.

### 4.2. Calibration Procedure

NSC is selected as the calibration dataset. Each stress–strain curve is first normalized as

(24) $$x=\frac{\varepsilon }{\varepsilon_{p}},\;y=\frac{\sigma }{f_{c}}$$

where *f*_c_ and *ε*_p_ are the peak stress and peak strain of the corresponding curve. Equation (13) is then calibrated branch by branch. For the ascending branch (*x* ≤ 1), *α*_a_ is obtained by least-squares fitting subject to *α*_a_ ≥ 1, so that the initial tangent modulus is not smaller than the secant modulus at the peak. For the descending branch (*x* > 1), *α*_d_ is fitted using

(25) $$y=\frac{x}{\left[\alpha d{\left(x-1\right)}^{2}+x\right]}$$

For the temperatures with a measured descending branch, the coefficient of determination of the full curve is *R*^2^ = 0.94–0.99. R^2^ was calculated using the normalized stress values over all available digitized points, including both the ascending and descending branches when the descending branch was available. Because the source study provides representative stress–strain curves rather than specimen-level raw observations, statistically meaningful experimental confidence bands cannot be reconstructed. The digitized points along each curve are correlated locations on the same stress–strain response rather than independent experimental replicates. Confidence bands estimated directly from their residuals would therefore represent only digitization and curve-fitting dispersion and could substantially underestimate the underlying experimental variability. For the curve at 200 °C, only the ascending branch is calibrated because the descending branch is unavailable. The temperature factors *k*_f_ and *k*_ε_ are obtained as the ratios of the peak stress and peak strain at each temperature to their values at 23 °C. The initial modulus *E*_0_ is not back-calculated from the initial slope of the digitized curves, because the data points in the initial segment are sparse and noisy and the resulting error may reach several times the true value. Instead, *E*_0_ is implicitly determined by the calibrated shape of the ascending branch, thereby maintaining the consistency relation

(26) $$\alpha_{a}=\frac{E_{c}\varepsilon_{p}}{f_{c}}$$

### 4.3. Calibration Results

**Figure 4:**
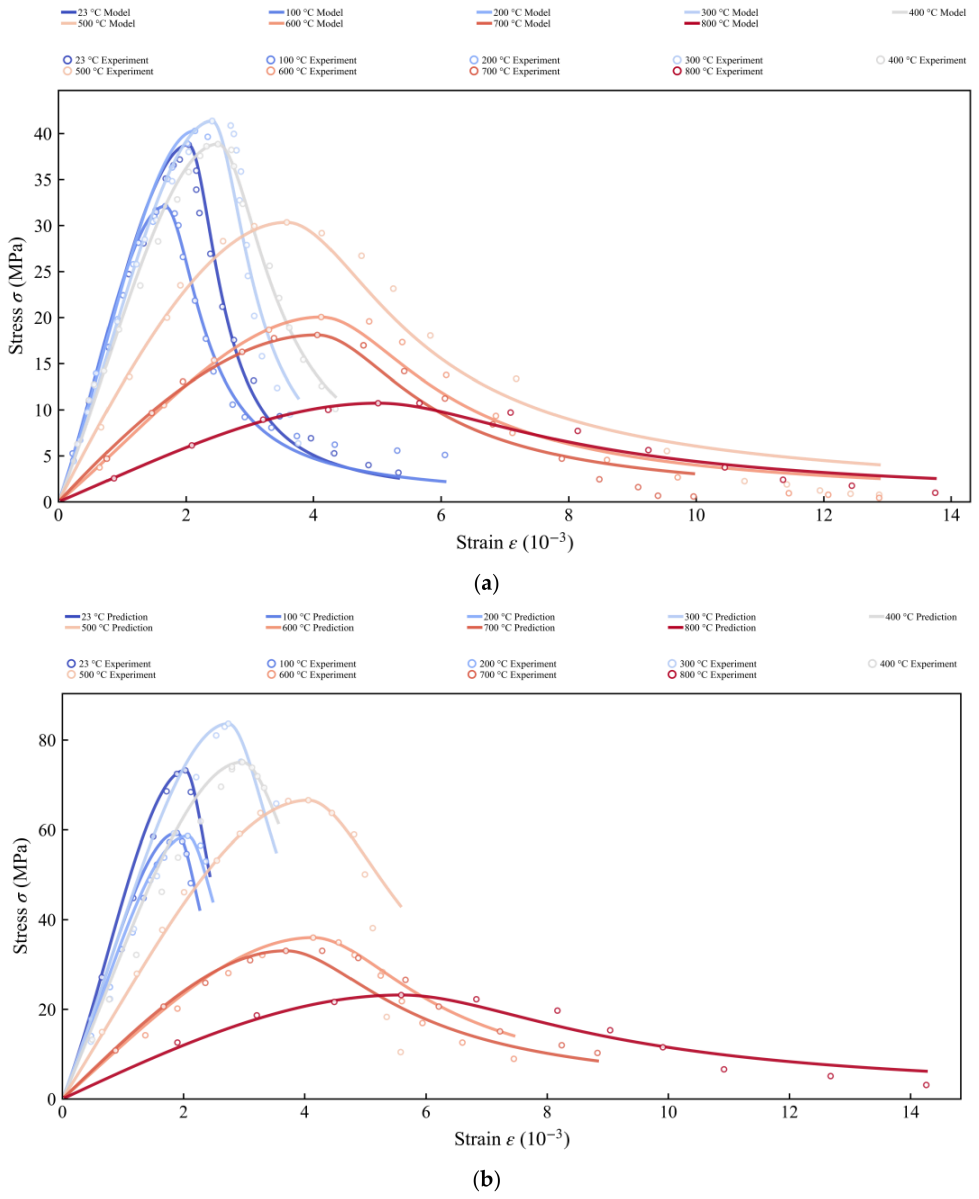

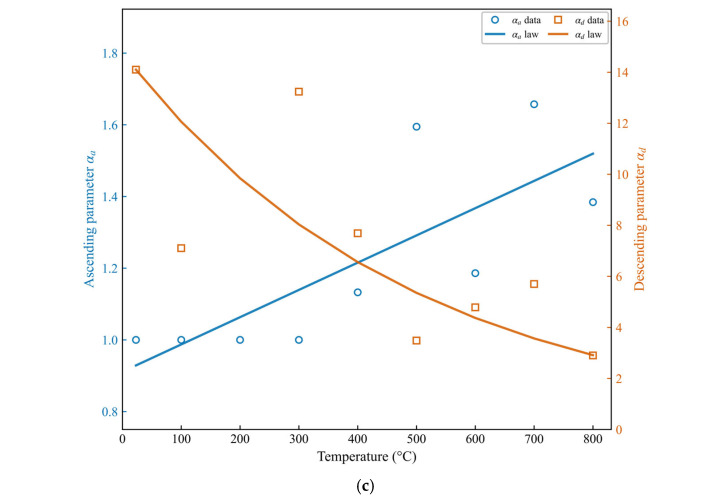
Calibration and validation of the high-temperature compressive model. (**a**) NSC calibration: complete stress–strain curves with experimental data (symbols) and model (lines), including post-peak softening. (**b**) HSC blind prediction: complete curves obtained by applying the NSC-calibrated shape laws to HSC’s own peak stress and strain (lines), compared with the HSC data (symbols), without refitting any shape parameter to HSC. (**c**) Temperature variation in the ascending parameter *α*_a_ and the descending parameter *α*_d_ (symbols, NSC fit; lines, calibrated laws).

The strength factor *k*_f_(*T*) exhibits a non-monotonic temperature dependence. It decreases slightly to about 0.83 at 100 °C, recovers over 200–400 °C and exceeds the room-temperature value, reaching about 1.07 at 300 °C, and then degrades substantially to about 0.28 at 800 °C. The entire curve lies above the Eurocode 2 reduction curve for siliceous aggregate concrete and approaches it only near 800 °C (Figure 3c). The underlying mechanism is discussed in Section 6.2. The peak-strain factor is expressed as

(27) $$k\varepsilon =1+0.06\xi +1.43\xi_{2},\;\;\;\;\xi =\frac{T-23}{777}$$

which gives *k*_ε_ ≈ 2.48 at 800 °C—an increase much smaller than the roughly tenfold increase assumed in Eurocode 2 (Figure 3d). The ascending-branch parameter *α*_a_(*T*) increases from about 1.0 at 23 °C to about 1.4–1.7 at elevated temperatures, indicating an increasingly rounded ascending branch and greater pre-peak nonlinearity (the implied *E*_0_ is about 19 GPa at 23 °C and about 3 GPa at 800 °C). The descending-branch, or damage-related, parameter *α*_d_(*T*) decreases from about 14.1 at 23 °C to about 2.9 at 800 °C, a trend described by

(28) $$\frac{\alpha_{d}\left(T\right)}{\alpha_{d}\left(23\right)}=e^{-0.00203\left(T-23\right)}$$

indicating that, after heating, compressive damage develops more slowly, post-peak softening becomes more gradual, and ductility increases. The quadratic form in Equation (27) was selected because the measured peak strain increases slowly at relatively low temperatures and more rapidly at higher temperatures. It is the lowest-order polynomial capable of representing this accelerating trend while ensuring that the peak-strain factor equals unity at the reference temperature of 23 °C. The exponential form in Equation (28) was selected because $\alpha_{d}$ remains positive and decreases approximately monotonically with temperature. This form represents a progressive proportional reduction and avoids the nonphysical negative values that may result from linear extrapolation. Both expressions are parsimonious empirical approximations to the available data rather than universal temperature relationships for concrete. Scatter exists over 200–400 °C, corresponding to the moisture-related anomaly in this interval (Figure 4a,c).

### 4.4. Validation Results

The high-temperature regime provides the strongest validation in this study. Because the dataset is based on material strain and includes the descending branch, the model directly reproduces the complete NSC stress–strain curves from 23 to 800 °C, including both the ascending and softening branches, with *R*^2^ = 0.94–0.99 (Figure 4a). In this case, the compressive damage variable and *α*_d_ are calibrated from the measured softening branch rather than introduced as assumptions, in contrast to the low-temperature regime of Section 3.

The normalized parameter-evolution laws calibrated solely on NSC are then transferred to HSC. Using HSC’s measured peak stress and peak strain at each temperature together with the shape laws *α*_a_(*T*) and *α*_d_(*T*), the complete HSC stress–strain curves are predicted without refitting any shape parameter to HSC (Figure 4b). The prediction reproduces the HSC curves with *R*^2^ ≥ 0.91 at eight of the nine temperatures (median about 0.94); the only notable exception is 500 °C (*R*^2^ ≈ 0.66), where the measured HSC softening departs from the NSC-calibrated trend. Because HSC differs substantially in strength from NSC and none of the HSC data were used in the calibration, this constitutes a genuine, partially independent cross-strength-grade validation. It is further supported by the close agreement of the measured *k*_f_ and *k*_ε_ of HSC with those of NSC and with the calibrated laws (Figure 3c,d). Together, these results indicate that the normalized laws governing temperature evolution, covering both the strength and ductility factors and the curve shape parameters, are transferable between normal-strength concrete and high-strength concrete.

## 5. Assessment of the Three-Dimensional Extension Under Multiaxial Compression

Section 2.7 extends the uniaxial damage constitutive model to a three-dimensional formulation. In this section, high-temperature triaxial test data are used to validate this extension from two aspects: the ability of the yield surface to describe multiaxial strength (Section 5.1), and the ability of the equivalent uniaxial skeleton curve to represent multiaxial stress–strain responses (Section 5.2). The validation data are taken from the true-triaxial tests on plain high-strength concrete HSC60 reported by He et al. [26]. The specimens were exposed to 20, 200, 300, 400, 500, and 600 °C, cooled to room temperature, then tested in the residual state under six triaxial stress ratios (plus the uniaxial baseline, seven in total), ranging from $\sigma_{1}:\sigma_{2}:\sigma_{3}=0:0:-1$ to −0.10:−1:−1, with $\sigma_{1}/\sigma_{3}$ generally kept at 0.1 in the triaxial series. The three principal failure strengths, peak strains, and complete stress–strain curves were reported in Table 2 and Figure 8 of [32].

It should first be clarified that these data correspond to residual HSC60, whereas the uniaxial NSC data used in Section 4 were obtained under hot-state conditions. The two datasets therefore differ in both thermal condition and concrete type. In this section, the residual uniaxial strength $f_{c}\left(T\right)$ and peak strain measured from the same batch of specimens by He et al. are used as the normalization basis. Consequently, the validation is internally self-consistent within the same dataset. What is examined here is the shape of the yield surface and the equivalent skeleton curve, together with their evolution with temperature, rather than the absolute strength level. This treatment is independent of the hot-state uniaxial calibration in Section 4. Given the current lack of multiaxial data covering the broad temperature range for a single concrete under identical testing conditions, this provides a feasible and rigorous normalized validation of the model shape.

### 5.1. Three-Dimensional Yield Surface and Multiaxial Strength Validation

The yield surface used in the three-dimensional extension adopts the Lubliner–Lee–Fenves form [15,16]. In the effective stress space, it is written as

(29) $$F=\frac{1}{1\;-\;\alpha }\left(\alpha {\bar{I}}_{1}+\sqrt{3{\bar{J}}_{2}}+\beta \langle {\bar{\sigma }}_{max}\rangle -\gamma \langle -{\bar{\sigma }}_{max}\rangle \right)-{\bar{c}}_{c}\left(\kappa_{c}\right)\leq 0$$

where ${\bar{I}}_{1}=tr\bar{\sigma }$ and ${\bar{J}}_{2}=\frac{1}{2}\bar{s}:\bar{s}$ are the first invariant and the second deviatoric invariant of the effective stress, respectively; ${\bar{\sigma }}_{max}$ is the maximum principal effective stress; and ⟨⋅⟩ denotes the Macaulay bracket. The parameter α is determined by the ratio between the equibiaxial compressive strength $f_{b0}$ and the uniaxial compressive strength $f_{c0}$:

(30) $$\alpha =\frac{f_{b0}/f_{c0}-1}{2f_{b0}/f_{c0}-1}$$

Taking $f_{b0}/f_{c0}=1.16$ gives α=0.121. The parameter β is determined by the tensile and compressive strengths, whereas γ is determined by the ratio between the tensile and compressive meridians, $K_{c}$, as

(31) $$\gamma =\frac{3\left(1\;-{\;K}_{c}\right)}{2K_{c\;}-\;1}$$

Under uniaxial compression, the maximum principal effective stress is zero, and Equation (29) degenerates into Equation (11), thereby ensuring consistency between the uniaxial and three-dimensional formulations.

**Figure 5:**
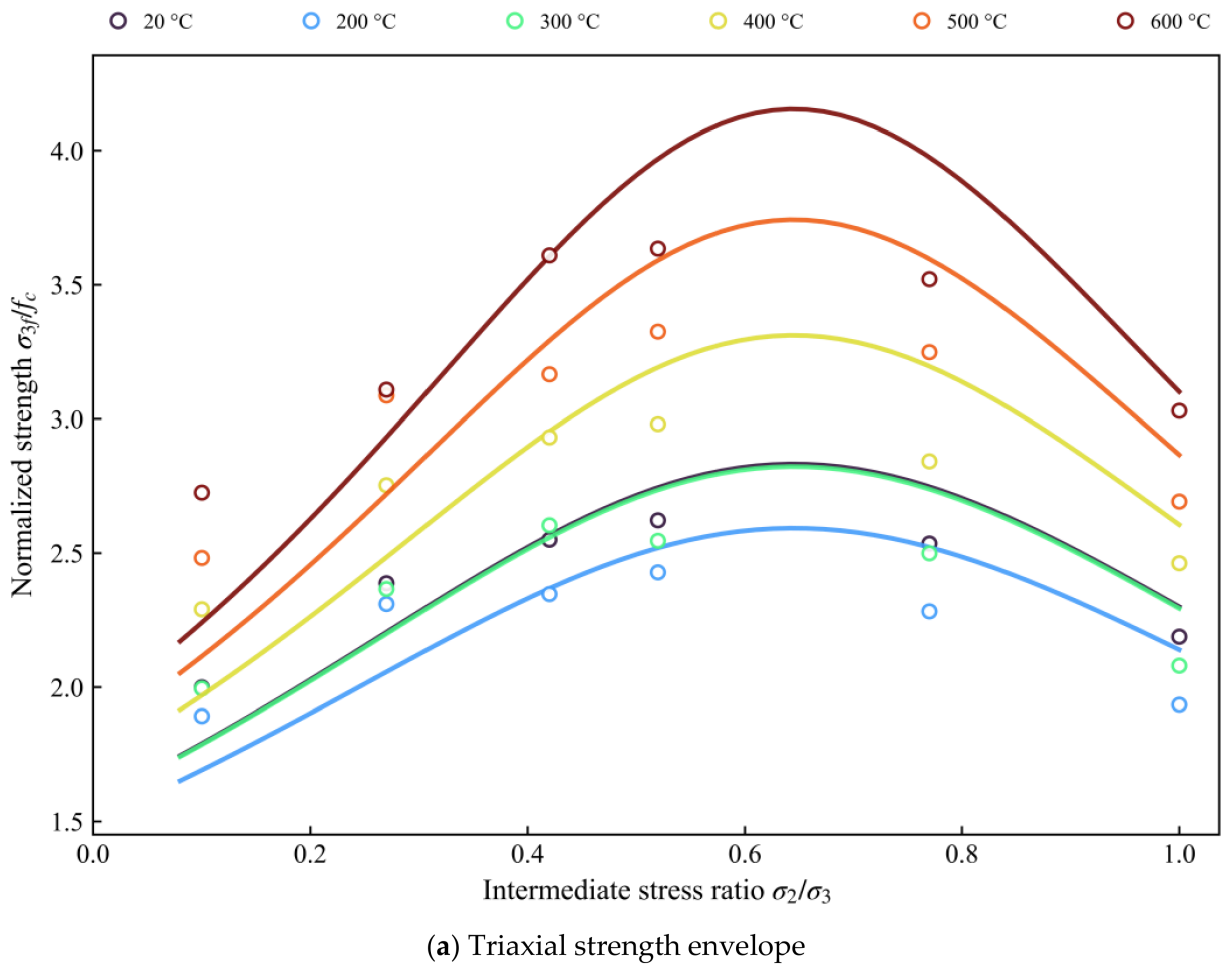

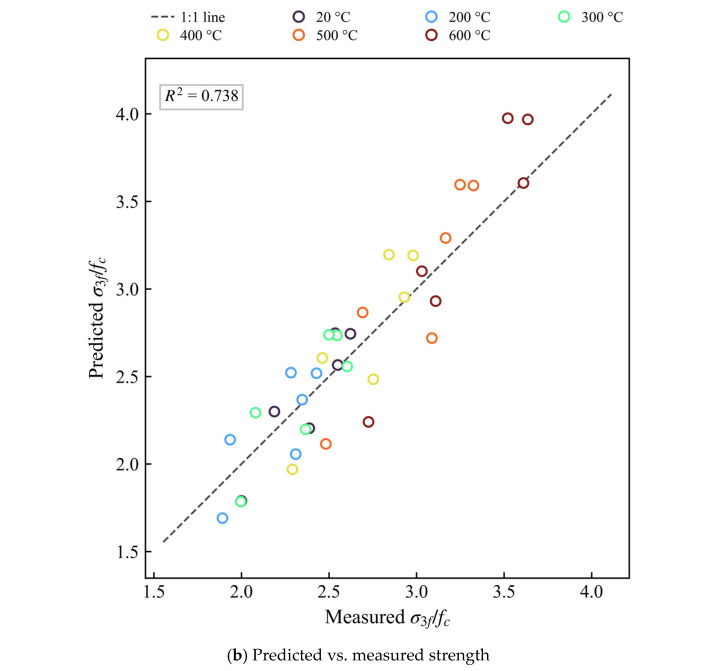
Multiaxial compressive strength validation: comparison between the yield surface in Equation (29), with α=0.121 and temperature-calibrated γ, and the residual HSC60 triaxial tests of He et al. [32] at 20–600 °C. (**a**) Variation in the triaxial strength envelope $\sigma_{3f}/f_{c}$ with the intermediate stress ratio $\sigma_{2}/\sigma_{3}$; symbols denote tests and lines denote model predictions at different temperatures. (**b**) Predicted versus measured triaxial strengths for 36 data points.

For proportional loading with $\sigma_{1}:\sigma_{2}:\sigma_{3}=r_{1}:r_{2}:-1$, setting F=0 in Equation (29), with the effective compressive strength equal to $f_{c}\left(T\right)$ and the maximum principal effective stress satisfying ${\bar{\sigma }}_{max}=\sigma_{1}<0$, gives the failure strength in the major compressive direction as

(32) $$\frac{\left|\sigma_{3f}^{T}\right|}{f_{c}\left(T\right)}=\frac{1\;-\;\alpha }{\sqrt{\frac{1}{2}\left[{\left(r_{1}\;-\;r_{2}\right)}^{2}\;+\;{\left(r_{2}\;-\;1\right)}^{2}\;+\;{\left(1\;-\;r_{1}\right)}^{2}\right]}\;-\;\alpha \left(r_{1}\;+\;r_{2}\;+\;1\right)\;-\;\gamma r_{1}}$$

where $r_{1}=\sigma_{1}/\sigma_{3}$ and $r_{2}=\sigma_{2}/\sigma_{3}$. The parameter α is fixed at 0.121, corresponding to a biaxial-to-uniaxial compressive strength ratio of 1.16, because no biaxial data are available for independent calibration. Only γ is calibrated against the triaxial data at each temperature. The calibrated value of γ increases from approximately 2.6 at 20 °C to approximately 3.6 at 600 °C, corresponding to a decrease in $K_{c}$ from 0.68 to 0.65. With this temperature-dependent γ, Equation (29) predicts the 36 multiaxial strength points with an average relative error of approximately 8% (Figure 5).

The increase in γ with temperature indicates that the deviatoric shape of the yield surface evolves with temperature and that the compressive meridian expands relatively. This is consistent with the transition from localized damage to more diffuse damage at elevated temperatures, as discussed in Section 6.2. Diffuse microcracking weakens the dominant role of deviatoric stress in failure and leads to a relative expansion of the envelope along the compressive meridian. If the uncalibrated standard parameter set is used instead, namely γ=3 and $K_{c}=2/3$, the error increases to approximately 14%, with a systematic overestimation of the confinement benefit. This agrees with the conclusion of He et al. [32] that the triaxial-to-uniaxial strength ratio of HSC is lower than that of normal-strength concrete, indicating that the high-temperature multiaxial parameters should be calibrated against measured data.

Two limitations of this validation should be explicitly acknowledged. First, $\sigma_{1}/\sigma_{3}$ is nearly fixed at 0.1 for the triaxial stress ratios, and only $\sigma_{2}/\sigma_{3}$ varies. Therefore, α and γ are correlated in the fitting and cannot be independently identified. If both parameters are allowed to vary simultaneously, the fit can be improved to an error of approximately 5% ($R^{2}\approx 0.94$), but α tends toward zero, corresponding to $f_{b0}/f_{c0}\approx 1$, which is physically unreasonable. Therefore, this study retains the standard value of α and reports only $\gamma \left(T\right)$. Second, the validation involves compression only; tensile damage *d*_t_ and the tensile meridian controlled by beta are not examined. Nevertheless, Equation (29) reproduces the triaxial compressive strength envelope and its temperature evolution over 20–600 °C and six stress ratios with an accuracy of approximately 8%, thereby providing independent support for the three-dimensional strength surface associated with the proposed uniaxial damage model.

### 5.2. Compatibility of the Equivalent Uniaxial Skeleton Curve with Multiaxial Stress–Strain Responses

**Figure 6:**
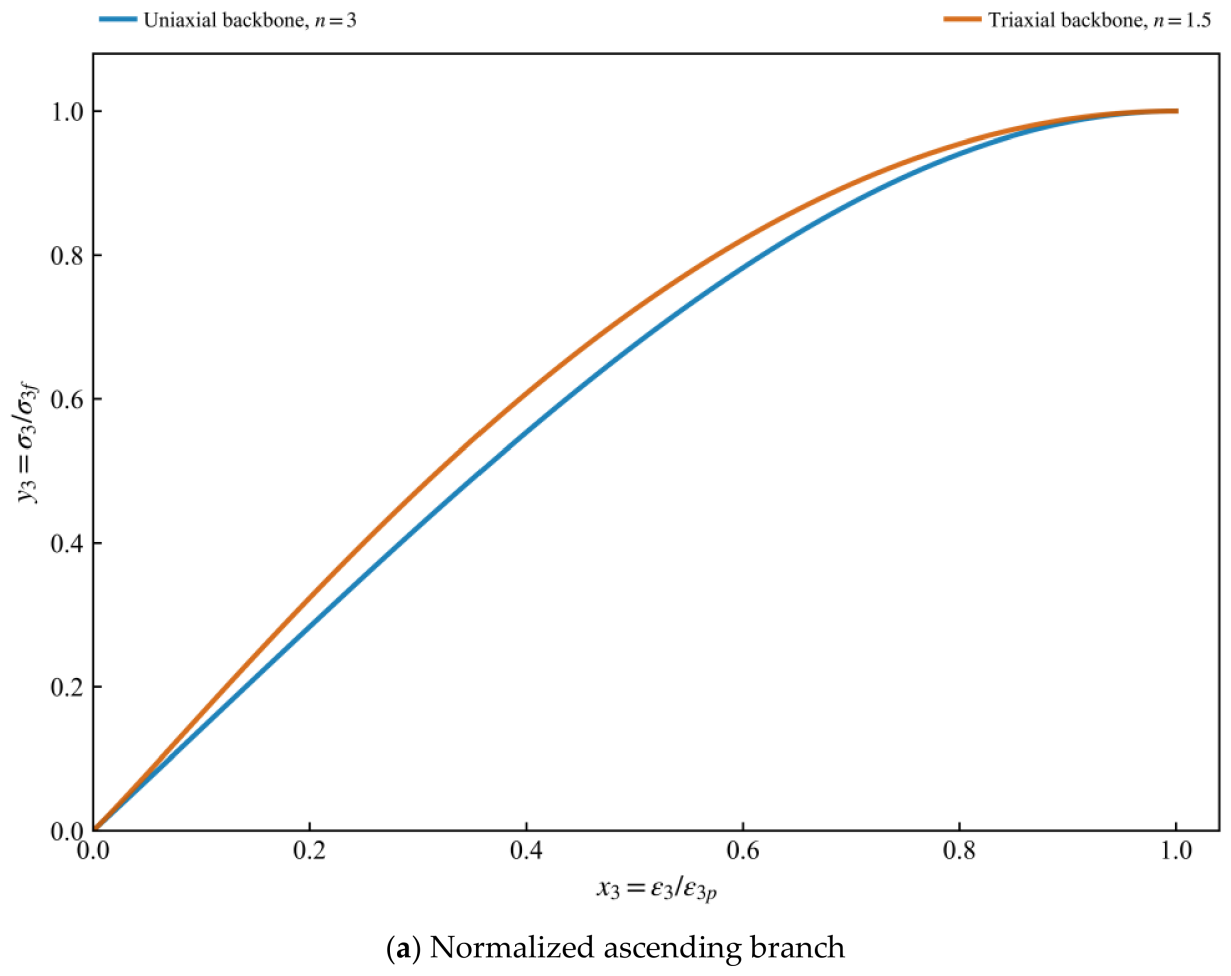

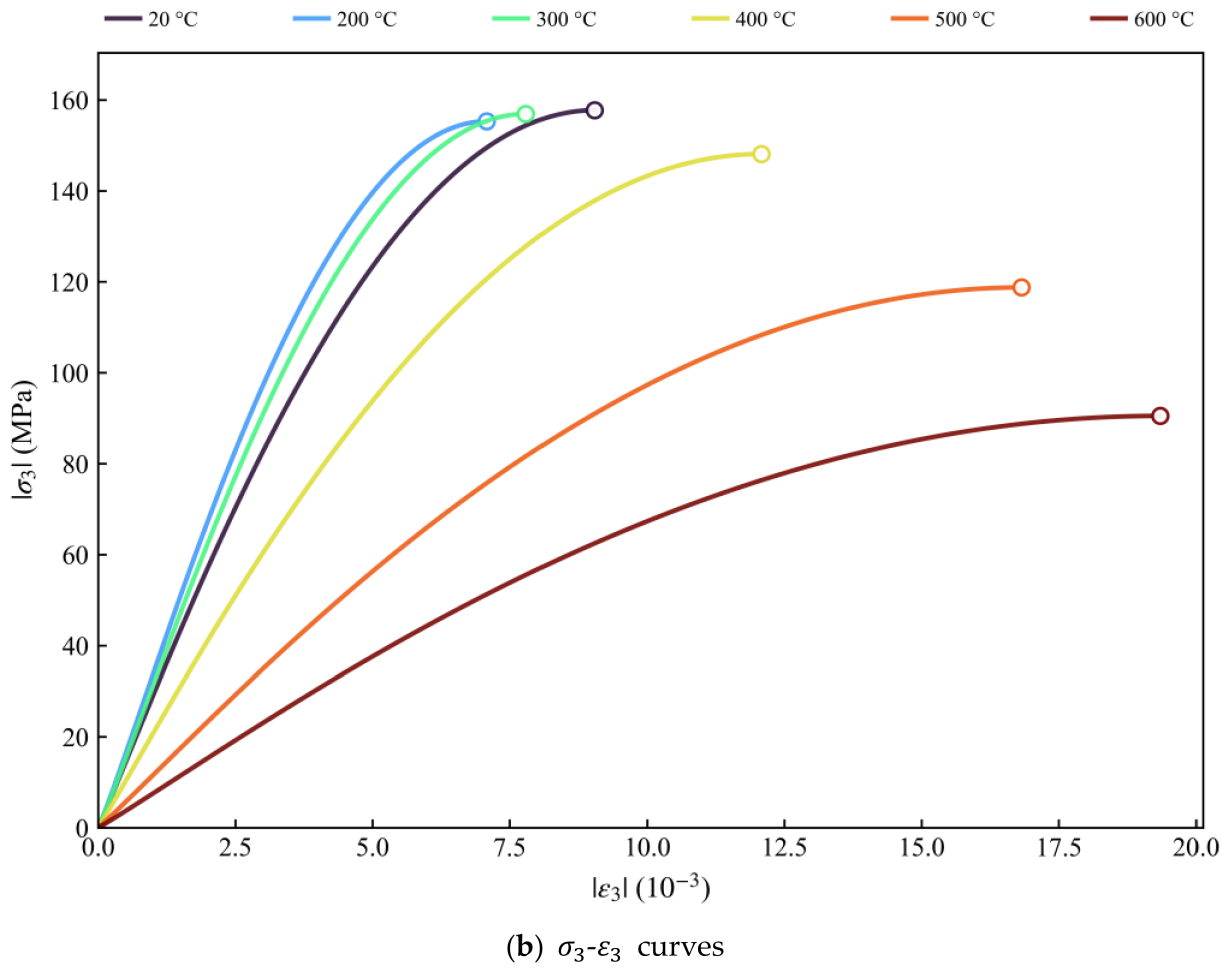
Multiaxial stress–strain curve validation, (**a**) comparing the normalized ascending branches with n≈1.5 and n=3 and (**b**) presenting the reconstructed $\sigma_{3}$-$\varepsilon_{3}$ curves at $\sigma_{2}/\sigma_{3}=0.52$ and 20–600 °C. Lines denote the reconstructed curves based on Equation (33) with n→1.5, and symbols denote the measured peak points reported by He et al. [32].

After the multiaxial failure strength is described by the yield surface, it is still necessary to examine whether the constitutive formulation can describe multiaxial deformation, namely the stress–strain curves. Within the equivalent uniaxial framework, the equivalent uniaxial curve in the major compressive direction, $\sigma_{3}$, follows the same skeleton-curve family as Equation (13), normalized by the multiaxial peak stress and peak strain in that direction, namely $\sigma_{3f}^{T}$ and $\epsilon_{3p}^{T}$. He et al. [32] measured complete triaxial stress–strain curves of HSC60 at different temperatures and stress ratios, with the experimental curves shown in their Figure 8. They further established a constitutive representation using the polynomial,

(33) $$y=Ax+Bx^{2}+Cx^{n}$$

which matched the experimental curves well [33]. In that formulation,

(34) $$A=\frac{E_{0}^{T}}{E_{if}}$$

which corresponds to $\alpha_{a}$ in Equation (13), and the exponent is taken as n=1.5 under triaxial compression. By comparison, the ascending branch of Equation (13) is the special case of the Guo–Xu polynomial with n=3, for which it degenerates into the cubic expression adopted in GB 50010 [33,34]. Thus, the skeleton curve used in this study and the curve adopted by He et al. belong to the same polynomial family; the main difference lies in the exponent of the ascending branch [32] (Figure 6a).

The triaxial compression curve with n≈1.5 is more rounded than the uniaxial curve with n=3, indicating more pronounced pre-peak plastic deformation. This is consistent with the experimental feature that increasing confinement enhances ductility and makes the peak less distinct. Using the values of $\alpha_{a}$ reported in Table 3 of He et al. [32], namely 1.50, 1.18, 1.22, 1.56, 1.48, and 1.45 from 20 to 600 °C, together with the measured peak values $\sigma_{3f}^{T}$ and $\varepsilon_{3p}^{T}$ in their Table 2, the reconstructed $\sigma_{3}$-$\varepsilon_{3}$ curves pass through the measured peak points and reproduce the main curve-shape features reported in Figure 8 of He et al. [32] over the full temperature range of 20–600 °C (Figure 6b). As temperature increases, the peak strain $\varepsilon_{3p}^{T}$ increases markedly, reaching more than twice the room-temperature value at 600 °C; the ascending branch becomes more rounded, and the post-peak softening becomes more gradual. These trends are consistent with the high-temperature uniaxial response identified in Section 4, where $k_{\varepsilon }$ increases and $\alpha_{d}$ decreases.

The limitations of this validation should also be stated. First, although Figure 8 of He et al. [32] presents measured stress–strain curves, the same coordinate system contains seven stress ratios multiplied by three strain components, resulting in 21 heavily overlapping curves. It is therefore difficult to perform robust pointwise digitization of individual $\sigma_{3}$-$\epsilon_{3}$ curves. Accordingly, this section validates the model using reliably extractable controlling features: the exact peak values reported in Table 2 of He et al. [32], the rounded ascending-branch shape, and the increase in peak strain with temperature. These features are reproduced by Equation (33) with n≈1.5. The good agreement obtained by He et al. [32] using the same polynomial family with n=1.5 to fit their Figure 8, as shown in their Figure 10 of He et al. [32], further supports the ability of this curve family to reproduce the measured curves, although this is not a pointwise fit to the original curves in the present study. Second, the multiaxial peak strain $\epsilon_{3p}^{T}$ is taken from the measurements. The present uniaxial damage framework does not yet provide a predictive equation for the multiaxial peak strain; its dependence on stress ratio and temperature. Third, as in Section 5.1, the validation is limited to residual HSC60 under compression.

Taken together, Section 5.1 and Section 5.2 show that the yield surface for strength and the equivalent uniaxial skeleton curve for deformation jointly support the extension of the proposed uniaxial damage model to high-temperature multiaxial compression. The strength surface gives an error of approximately 8%, and the reconstructed deformation curves are consistent with both the measured peak values and the observed curve shapes.

## 6. Discussion

### 6.1. Temperature-Dependent Micromechanisms and Constitutive Interpretation

Concrete is a solid–liquid–gas three-phase porous medium; therefore, its temperature-dependent mechanical response is governed by the evolution of pore water, hydration products, and thermally induced microcracking [1,3,7,13]. The low-temperature and high-temperature regimes are controlled by fundamentally different mechanisms, which explains why the temperature effect cannot be represented by a single monotonic empirical function.

In the sub-zero regime, the dominant mechanism is the freezing of pore water. Near 0 °C, pore water has not yet fully frozen, and cooling mainly induces slight thermal contraction. Liquid water does not contribute to load-bearing, while minor thermal mismatch between cement paste and aggregates may generate limited microcracking. This explains the strength valley around 0 °C, where the strength factor $k_{f}$ reaches its minimum. Below 0 °C, pore water progressively freezes from larger pores to smaller pores because the freezing point of confined pore water is depressed by the Gibbs–Thomson effect. The formed ice fills and constrains the pore space and participates in load transfer with the solid skeleton, thereby increasing the effective load-bearing area and reducing stress concentration. As a result, compressive strength and stiffness increase. Meanwhile, the ice phase and the low-temperature cementitious matrix make the material more brittle, leading to a reduction in peak strain, a more linear ascending branch, and faster post-peak softening. In the present model, this low-temperature strengthening is treated as a reversible mechanism and is therefore incorporated into $E_{0}\left(T\right)$ and the strength threshold $r_{0}^{-}$, rather than into damage. This treatment is consistent with the experimental observation that low-temperature strengthening can largely recover after rewarming and reappear upon subsequent cooling [1].

In the high-temperature regime, the governing mechanism changes from reversible pore-water freezing to irreversible dehydration and microcracking. At relatively low heating temperatures, evaporation of free and capillary water may reduce capillary and disjoining pressures, producing a slight strength decrease around 100 °C. In the range of approximately 200–400 °C, moisture migration, continued hydration of unhydrated cement, and possible re-densification of the C-S-H gel structure may lead to a temporary recovery of strength, corresponding to the non-monotonic increase in $k_{f}$. At higher temperatures, however, dehydration of C-S-H, dehydroxylation of Ca(OH)_2_, thermal incompatibility between aggregates and cement paste, and phase transformation or decomposition of aggregates progressively dominate the response. These processes generate distributed microcracking and irreversible degradation, leading to substantial reductions in strength and stiffness at 600–800 °C.

This mechanistic transition is directly reflected in the calibrated constitutive parameters. The thermal-mismatch microcracking at high temperature is represented by the irreversible thermal damage variable $d_{\theta }$ and is incorporated into the temperature factors of strength and elastic modulus, $k_{f}$ and $k_{E}$. Unlike the localized brittle splitting observed at low temperature, high-temperature damage is more diffuse, causing the descending branch to become more gradual, $\alpha_{d}$ to decrease, and ductility to increase. The marked increase in peak strain, represented by $k_{\varepsilon }$, is jointly associated with microcrack opening, stiffness degradation, and transient thermal creep. The same diffusion of damage also explains the relative widening of the compressive meridian of the yield surface with increasing temperature, as reflected by the increase in γ in Section 5.1.

Therefore, the proposed framework is not formulated as an unrelated collection of temperature-specific stress–strain curves. The calibrated parameter trends are physically interpreted in terms of the transition from reversible ice-induced strengthening at sub-zero temperatures to irreversible dehydration- and microcracking-induced degradation at elevated temperatures. This interpretation provides a mechanism-informed basis for modifying the stiffness and strength thresholds reversibly in the sub-zero regime and introducing irreversible thermal degradation at elevated temperatures. Nevertheless, because these parameter laws were calibrated using published macroscopic test data rather than directly identified from microstructural measurements, their mechanistic interpretation should be regarded as physically plausible rather than uniquely experimentally verified.

### 6.2. Unified Full-Range Behavior and Physical Meaning of Parameters

**Figure 7:**
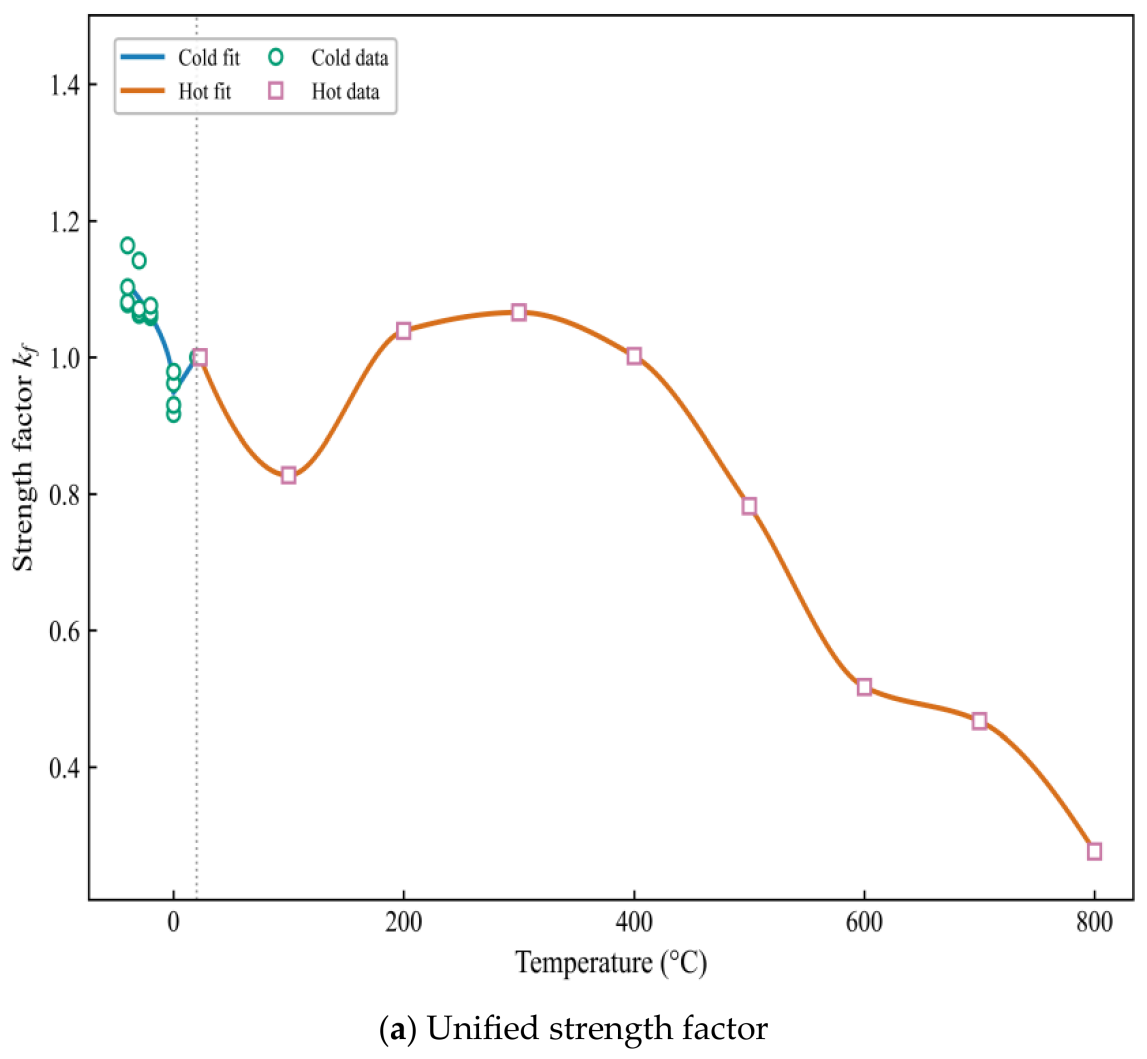

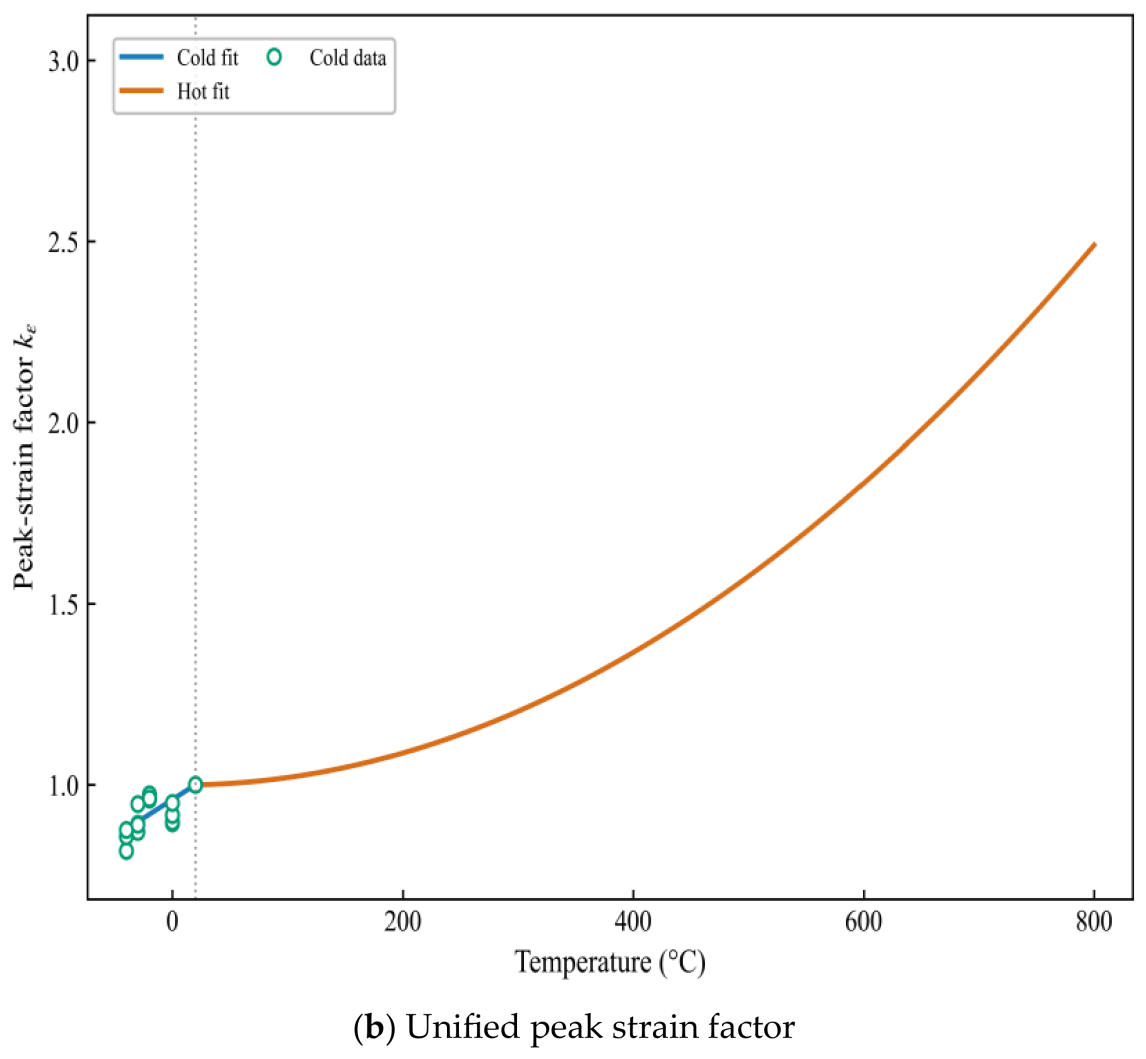
Unified parameter evolution laws over the range from −40 to 800 °C. The cold-end data are from Li et al. [9], and the hot-end data are from Kodur [3]; both are normalized by their respective room-temperature values.

By normalizing the parameter evolution laws for the cold and hot regimes separately and connecting them at approximately 20 °C, a unified evolution law over the range from −40 to 800 °C is obtained (Figure 7). The strength factor links three characteristic features: the freezing-point valley in the sub-zero regime, the moisture-related strength recovery above the Eurocode prediction in the range of 200–400 °C, and the pronounced degradation toward 800 °C. The peak-strain factor increases monotonically from the coldest state, where the material is most brittle, to the hottest state, where the material becomes most ductile.

**Figure 8:**
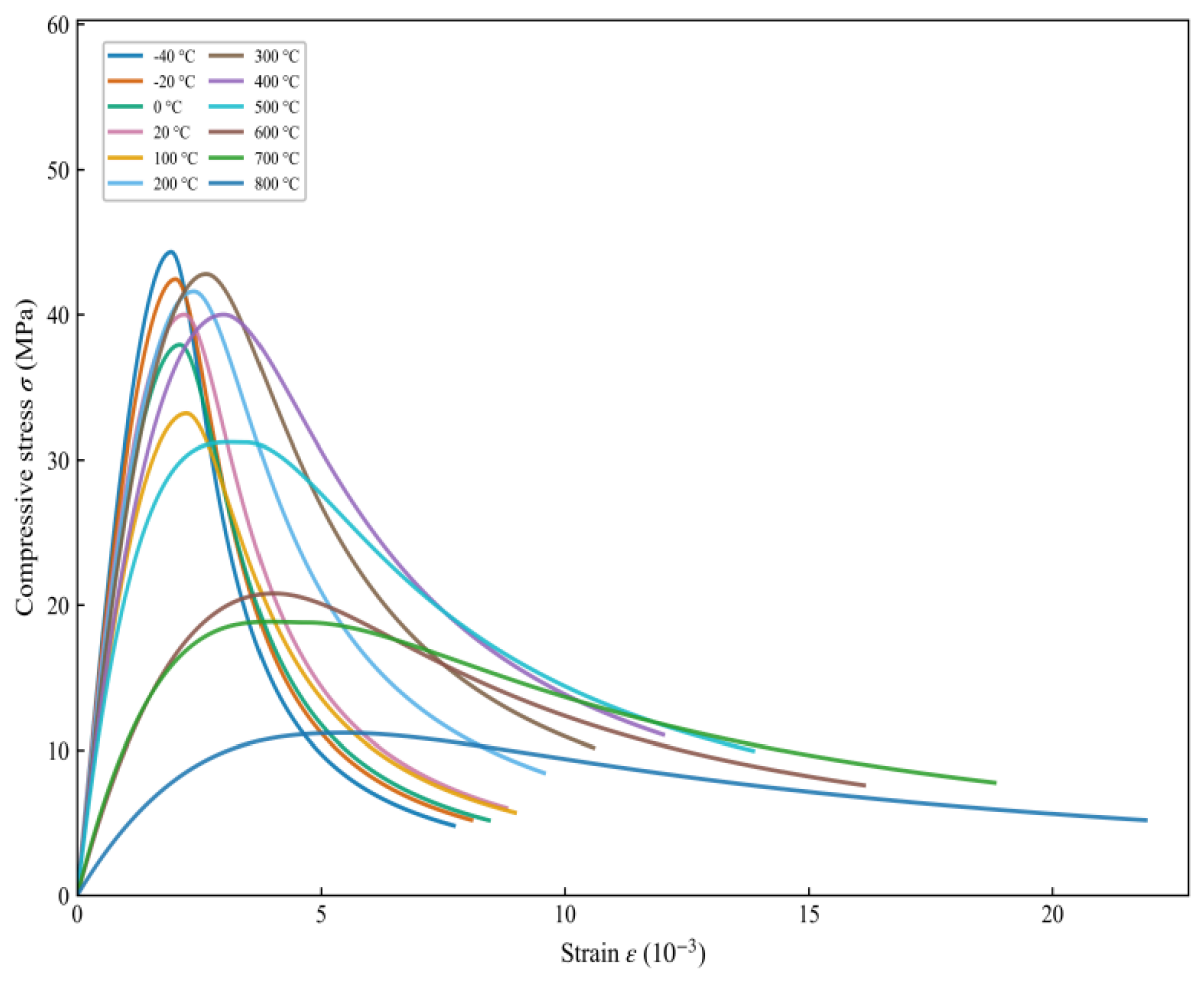
Family of stress–strain curves predicted by the unified model over the range from −40 to 800 °C. The reference concrete is defined by $f_{c0}$ = 40 MPa, $\epsilon_{p0}$ = 0.0022, $\alpha_{a0}$ =2.0, and $\alpha_{d0}$ = 2.5. The curves illustrate the response implied by the calibrated parameter-evolution laws and do not represent independent experimental predictions.

For a specified room-temperature reference set, namely $f_{c0}$, $\epsilon_{p0}$, $\alpha_{a0}$, and $\alpha_{d0}$, the calibrated parameter laws generate a continuous family of stress–strain curves over the broad temperature range considered in this study (Figure 8). These curves illustrate the constitutive implications of the fitted temperature functions and should not be interpreted as independently validated predictions for an arbitrary concrete mixture. In the low-temperature regime, the curves are higher and more brittle, with a larger $\alpha_{d}$ and sharper post-peak softening. In the high-temperature regime, the curves are lower and more ductile, with a smaller $\alpha_{d}$ and an elongated post-peak tail. This continuous transition from low-temperature embrittlement to high-temperature ductilization is consistent with the description of continuum damage–plasticity theory [15,16,21].

The four parameters have clear physical meanings and can be interpreted consistently through the underlying micromechanisms. The factor $k_{f}$ scales the peak strength and reflects the increase or loss of load-bearing phases, such as ice filling at low temperature or dehydration-induced decomposition at high temperature. The factor $k_{\epsilon }$ controls the peak-strain location and reflects the amount of irreversible deformation that can be accumulated; it is small at low temperature because of ice-induced constraint and large at high temperature because of microcracking and creep. The parameter $\alpha_{a}$ controls the curvature of the ascending branch and reflects the development of pre-peak plasticity or damage. The parameter $\alpha_{d}$ controls the softening rate and the growth of compressive damage, reflecting the degree of failure localization. It is large in the low-temperature regime because failure tends to occur by localized splitting, whereas it is small in the high-temperature regime because microcracking becomes more diffuse.

In terms of sensitivity, the model is most sensitive to $\alpha_{d}$ and $k_{f}$. The former governs post-peak energy dissipation and residual load-bearing capacity, whereas the latter scales the overall strength level. The model is relatively less sensitive to $\alpha_{a}$. The opposite trends of $\alpha_{a}$ and $\alpha_{d}$ on the two sides of room temperature, together with the monotonic increase in $k_{\epsilon }$, jointly reflect the essential full-range transition from low-temperature embrittlement to high-temperature ductilization.

### 6.3. Comparison with Existing Models and Limitations

The discrepancy between the present framework and Eurocode 2 [25] is mainly reflected in the predicted high-temperature strength and peak strain. First, the calibrated strength law exhibits a moisture-related recovery over approximately 200–400 °C, and the residual strength remains higher than the Eurocode 2 value up to approximately 700 °C. The calibrated strength is therefore generally higher than the code-based reduction curve, suggesting that Eurocode 2 is conservative for the specific normal-strength concrete dataset considered here. Second, the increase in peak strain is substantially smaller than that prescribed by Eurocode 2. At 800 °C, the peak strain represented by the adopted dataset is approximately 2.5 times its room-temperature value, whereas Eurocode 2 assumes an increase of approximately ten times. Direct application of the code-based relationship would therefore substantially overestimate the deformation represented by this dataset. These discrepancies demonstrate the importance of calibrating temperature-dependent parameters against material-specific measurements. However, they should not be generalized without qualification because the observed differences may depend on concrete strength, aggregate type, moisture condition, heating history, and testing procedure.

Compared with empirical formulations developed for a single temperature regime [14,23,35], the present framework retains one thermodynamically consistent constitutive structure across the sub-zero and elevated-temperature regimes and explicitly represents post-peak softening through the compressive damage variable. In terms of overall predictive scope, the framework occupies an intermediate position between design-oriented empirical models and fully coupled thermo-mechanical or hygro-thermo-mechanical formulations. Code-based and empirical models are generally simpler and more robust within their intended application ranges, but they often prescribe only selected temperature-dependent properties or stress–strain relationships and do not explicitly connect reversible sub-zero strengthening with irreversible high-temperature degradation. Fully coupled formulations can represent additional processes such as moisture transport, pore-pressure evolution, transient thermal strain, and thermally induced cracking, but they require more state variables, material parameters, and numerical implementation effort. The present framework instead provides continuous compressive stress–strain curves, including post-peak softening, over the calibrated interval from −40 to 800 °C using a relatively compact parameter set. This gives the fitted parameters a clearer mechanical interpretation, but the available results do not establish superior predictive accuracy over existing constitutive models because no blind comparison based on common independent datasets was conducted. The principal differences between the proposed framework and representative existing temperature-dependent constitutive approaches are summarized in Table 1.

Table 1 indicates that the principal contribution of the present framework is not a demonstrated improvement in predictive accuracy over every established model, but the use of a relatively compact and mechanically interpretable constitutive structure across both sub-zero and elevated-temperature regimes. Fully coupled models retain advantages when moisture transport, pore pressure, transient thermal strain, or explicit fracture evolution must be resolved.

All experimental data used for parameter calibration and model assessment were obtained from previously published studies, and no new experiments were conducted specifically for the present work. The low-temperature regime from −40 to 20 °C was calibrated using the C30–C50 data reported by Li et al. [9], whereas the high-temperature regime from 23 to 800 °C was calibrated primarily using the normal-strength concrete curves summarized by Kodur [3]. Because the fitted parameters and the subsequent comparisons partially rely on the same datasets, the results in Section 3 and Section 4 should be interpreted primarily as parameter calibration accompanied by internal consistency assessment rather than as fully independent validation.

Limited cross-dataset assessments were nevertheless conducted for selected components of the framework. The high-strength concrete curves summarized by Kodur [3] were used to examine whether the normalized ascending- and descending-branch shape laws calibrated using normal-strength concrete could be transferred across strength grades. However, the measured peak stress and peak strain of the high-strength concrete were supplied before the normalized curve shapes were evaluated. This comparison therefore assesses the cross-strength-grade applicability of the normalized shape laws but does not constitute a blind prediction of the absolute high-strength concrete response. Similarly, the residual HSC60 true-triaxial data reported by He et al. [32] were used to assess the normalized multiaxial strength envelope and equivalent uniaxial curve shape. Because selected three-dimensional parameters were determined using the same dataset and the assessment was restricted to residual compression-dominated behavior, the results should be regarded as an assessment of selected three-dimensional components rather than as complete independent validation of the full damage–plasticity formulation.

The use of different literature datasets was necessary because a complete stress–strain dataset for the same concrete mixture over the entire interval from sub-zero to elevated temperatures is currently unavailable. The experimental requirements of the two regimes also differ substantially. Sub-zero strengthening depends on the presence and freezing of pore water, whereas high-temperature testing of highly saturated concrete may increase pore-pressure buildup and the risk of spalling [1,13]. The cold- and high-temperature datasets were therefore normalized using their respective room-temperature reference values and connected near room temperature. Consequently, the most defensible transferable quantities are the normalized parameter-evolution trends rather than the absolute strength, stiffness, strain, or damage parameters of a particular concrete mixture. For independent datasets with different concrete mixtures, moisture conditions, or aggregate types, the framework may retain the qualitative form of the normalized temperature trends, but its quantitative accuracy cannot be assumed. Changes in pore structure, freezable-water content, aggregate–paste thermal incompatibility, and initial mechanical properties may alter the strength, stiffness, peak-strain, and post-peak parameter laws. Accordingly, independent assessment and, where necessary, mixture-specific recalibration are required before application to substantially different concretes.

Several additional limitations arise from the available data and the adopted model scope. First, the low-temperature curves reported by Li et al. [9] are based on machine-displacement, or apparent, strain and do not contain descending branches. The material-level peak strain was therefore reconstructed by separating the estimated specimen response from machine compliance and platen-seating deformation, while the cold-regime post-peak parameter in Equation (23) was introduced as a physically motivated predictive prior rather than as a directly calibrated quantity. Second, neither the low- nor high-temperature dataset contains cyclic unloading–reloading data, so the separation between plastic strain and compressive damage relies on the closure assumption adopted in the constitutive formulation. Third, moisture content and degree of saturation are not treated as independent state variables, and their effects are represented only implicitly through the datasets used for calibration. Fourth, tensile damage, the tensile meridian, dilatancy, transient thermal strain, multiaxial peak-strain evolution, and hot-state multiaxial behavior remain unverified. The predictive capability of the framework beyond the concrete mixtures, moisture conditions, thermal histories, and compression-dominated loading paths represented by the source datasets has therefore not been established. Fully independent validation will require experiments on the same concrete mixture over the complete temperature interval, including controlled moisture conditions, direct material-strain measurements, complete low-temperature post-peak curves, cyclic unloading–reloading tests, tensile tests, and hot-state multiaxial tests.

## 7. Conclusions

This study developed a temperature-dependent thermo-elastoplastic damage framework for concrete over the calibrated temperature interval from −40 to 800 °C. By introducing temperature-dependent material parameters into an energy-based plasticity–damage formulation, the same constitutive structure was retained for both sub-zero and elevated-temperature regimes. All experimental data used for parameter calibration and model assessment were obtained from previously published studies, and no newly generated experiments were conducted. The reported comparisons should therefore be interpreted as parameter calibration, internal consistency assessment, and limited cross-dataset assessment of selected model components rather than as fully independent validation of the complete constitutive framework. The main conclusions are as follows.

(1) A unified modeling strategy was established by distinguishing reversible sub-zero strengthening from irreversible high-temperature degradation. In the sub-zero regime, ice-induced pore filling, confinement, and load transfer were represented through reversible modifications of the elastic stiffness and strength threshold. In the high-temperature regime, dehydration, thermal incompatibility, and thermally induced microcracking were represented phenomenologically through irreversible degradation of temperature-dependent material properties. This distinction allows the same elastoplastic damage structure to describe low-temperature embrittlement and high-temperature ductilization without treating the two regimes as unrelated empirical problems.
(2) The low-temperature calibration based on the C30–C50 concrete data showed that compressive strength and elastic modulus generally increase as temperature decreases below 0 °C, whereas the material peak strain decreases. The fitted strength factor represented the strength valley near the freezing point and the subsequent recovery at lower temperatures. However, the original low-temperature curves were expressed in terms of apparent machine-displacement strain and did not contain descending branches. The material peak strain was therefore reconstructed by separating the estimated specimen response from machine compliance and platen seating, while the cold-regime post-peak damage parameter was introduced as a physically motivated prior rather than being directly calibrated. Consequently, the predicted low-temperature post-peak response should be regarded as an extrapolated damage description, not as an experimentally validated softening law.
(3) The high-temperature calibration based on the normal-strength concrete curves provided the strongest material-level assessment in this study. The fitted framework reproduced the complete compressive stress–strain curves from 23 to 800 °C, including the post-peak branches, with R^2^ values of 0.94–0.99. The calibrated strength factor exhibited non-monotonic evolution, including a temporary recovery over approximately 200–400 °C followed by substantial degradation at higher temperatures. Meanwhile, the increase in peak strain and the decrease in the descending-branch parameter indicated a transition from relatively brittle and localized damage toward more ductile and diffuse deformation as temperature increased. When the normalized curve-shape laws calibrated using normal-strength concrete were applied to high-strength concrete after supplying its measured peak stress and peak strain, R^2^ values of at least 0.91 were obtained at eight of the nine temperatures. This result supports the cross-strength-grade transferability of the normalized curve-shape laws under the represented conditions, but it does not constitute a blind prediction of the absolute high-strength concrete response.
(4) The proposed temperature-dependent parameter laws provide continuous compressive stress–strain curve families from −40 to 800 °C based on a specified room-temperature reference state. The parameters retain identifiable mechanical meanings: the strength factor governs load-bearing capacity, the peak-strain factor characterizes deformation capacity, the ascending-branch parameter controls pre-peak nonlinearity, and the descending-branch parameter governs post-peak softening and damage-growth rate. From an engineering perspective, the framework provides a consistent material-input strategy for preliminary numerical analyses of LNG containment structures, cold-region concrete infrastructure, fire-exposed buildings, nuclear containment structures, and other concrete components subjected to extreme temperatures. Room-temperature material properties can be used as the reference state, while the proposed temperature functions update strength, stiffness, peak strain, and curve shape without switching between unrelated low- and high-temperature constitutive models. This may facilitate comparative assessment of temperature-induced changes in load-bearing capacity, deformation demand, damage localization, and residual performance under different thermal scenarios. However, mixture-specific calibration, moisture-state characterization, regularization of strain softening, and independent structural-scale validation remain necessary before the framework is used for design verification or safety assessment.
(5) Selected components of the three-dimensional extension based on the Lubliner–Lee–Fenves yield surface were assessed using published residual HSC60 data under true-triaxial compression after high-temperature exposure. With the biaxial compression parameter fixed and the temperature-dependent compressive-meridian parameter determined from the available data, the normalized multiaxial strength envelope was represented with an average relative error of approximately 8%. The corresponding equivalent uniaxial skeleton curves were also consistent with the main reported deformation characteristics under triaxial compression, including increased peak strain and a more rounded ascending branch. Nevertheless, because selected three-dimensional parameters were determined using the same dataset and only residual compression-dominated behavior was considered, this comparison should be regarded as a normalized multiaxial assessment rather than as complete independent validation of the full three-dimensional damage–plasticity formulation.
(6) Important limitations remain. The sub-zero and elevated-temperature regimes were calibrated using different concrete datasets; consequently, the quantities most reasonably transferred across regimes are the normalized parameter-evolution trends rather than the absolute material properties. The calibrated temperature functions are also conditional on the concrete compositions, moisture states, thermal histories, and loading conditions represented by the source datasets. In addition, the low-temperature descending branch, unloading–reloading response, tensile damage, dilatancy, transient thermal strain, multiaxial peak-strain evolution, and hot-state multiaxial behavior were not directly validated. The current results therefore demonstrate the internal consistency and limited cross-dataset applicability of the proposed framework but do not establish unrestricted predictive capability. Future work should conduct full-temperature-range experiments on the same concrete mixture under controlled moisture conditions, with particular emphasis on direct material-strain measurements, low-temperature post-peak compression, cyclic unloading–reloading, tensile loading, and hot-state multiaxial testing.

## Figures and Tables

**Table 1:** Qualitative comparison of representative temperature-dependent concrete models.

Model or Approach	Main Assumptions and Features	Temperature Range and Validation	Applicability and Limitations
High-temperature empirical models [22,23]	Temperature-dependent uniaxial stress–strain laws; selected models include transient thermal deformation	Elevated temperatures; calibrated or compared with uniaxial test data	Simple and suitable for fire analysis, but limited to the high-temperature regime
Thermo-mechanical damage model [24]	Couples temperature-dependent plasticity and damage degradation	Elevated temperatures; assessed using material tests and numerical applications	Represents irreversible thermal damage but requires more complex implementation
Eurocode 2 [25]	Prescribed reduction factors and stress–strain relationships for design	Structural-fire temperatures; based on compiled experimental evidence	Convenient for engineering design but does not explicitly model damage evolution or sub-zero strengthening
Recent coupled models [26,27,28,29,30,31]	Include selected moisture transport, pore pressure, fracture, or multiscale mechanisms	Mainly elevated-temperature or application-specific conditions	Greater physical detail but more parameters and computational cost
Present framework	Effective-stress plasticity and energy-based damage; reversible sub-zero strengthening and irreversible high-temperature degradation	−40 to 800 °C; literature-based calibration and limited cross-dataset assessment	Compact full-range formulation, but requires mixture-specific recalibration and independent validation

## Data Availability

The raw data supporting the conclusions of this article will be made available by the authors on request.
